# Transcriptome profiles of *Quercus rubra* responding to increased O_3_ stress

**DOI:** 10.1186/s12864-020-6549-5

**Published:** 2020-02-14

**Authors:** Nourolah Soltani, Teo Best, Dantria Grace, Christen Nelms, Ketia Shumaker, Jeanne Romero-Severson, Daniela Moses, Stephan Schuster, Margaret Staton, John Carlson, Kimberly Gwinn

**Affiliations:** 1 The Department of Entomology and Plant Pathology, University of Tennessee, Knoxville, TN 37996 USA; 2 The Department of Ecosystem Science and Management, Pennsylvania State University, University Park, PA 16802 USA; 3 Department of Biological & Environmental Sciences, University of West Alabama, Livingston, AL 35470 USA; 4 Department of Biological Sciences, Notre Dame University, 46556 Notre Dame, IN France; 5 Singapore Centre for Environmental Life Sciences Engineering (SCELSE) Nanyang Technological University, Nanyang Avenue, 637551 Singapore

**Keywords:** Northern red oak, Transcriptome, Plant-pathogen interactions, Terpenoid, Mevalonic acid, Methylerythritol phosphate

## Abstract

**Background:**

Climate plays an essential role in forest health, and climate change may increase forest productivity losses due to abiotic and biotic stress. Increased temperature leads to the increased formation of ozone (O_3_). Ozone is formed by the interaction of sunlight, molecular oxygen and by the reactions of chemicals commonly found in industrial and automobile emissions such as nitrogen oxides and volatile organic compounds.

Although it is well known that productivity of Northern red oak (*Quercus rubra*) (NRO), an ecologically and economically important species in the forests of eastern North America, is reduced by exposure to O_3_, limited information is available on its responses to exogenous stimuli at the level of gene expression.

**Results:**

RNA sequencing yielded more than 323 million high-quality raw sequence reads. De novo assembly generated 52,662 unigenes, of which more than 42,000 sequences could be annotated through homology-based searches. A total of 4140 differential expressed genes (DEGs) were detected in response to O_3_ stress, as compared to their respective controls. Gene Ontology (GO) and Kyoto Encyclopedia of Genes and Genomes (KEGG) enrichment analyses of the O_3_-response DEGs revealed perturbation of several biological pathways including energy, lipid, amino acid, carbohydrate and terpenoid metabolism as well as plant-pathogen interaction.

**Conclusion:**

This study provides the first reference transcriptome for NRO and initial insights into the genomic responses of NRO to O_3_. Gene expression profiling reveals altered primary and secondary metabolism of NRO seedlings, including known defense responses such as terpenoid biosynthesis.

## Background

Northern red oak (*Quercus rubra* L.) (NRO), a monocoecious species belonging to Fagaceae family, is an ecologically and economically important forest tree in North America. It is a valuable source of hardwood lumber, often used for flooring, veneer and furniture for higher grade timber and for firewood for the lower grades [1, 2]. This hardwood species has a wide range of habitat from northern Ontario to southern Alabama and the Atlantic coast to Nebraska [3, 4]. NRO is the dominant tree species in many of the forest types across its native range, and NRO mast provides food for many native wildlife species [5–7]. NRO has a number of features that make it a good model for studies of population genetics, speciation and gene flow, including co-habitation and hybridization with several close congeners, an outcrossing mating system, and a wide geographical range [8–12].

NRO is impacted by oak population decline, a disease complex caused by a combination of biotic and abiotic stresses, originally described in the 1970’s in oak-dominated southeastern forests [13]. In 1999, oak decline had severely affected about 400,000 acres of forests throughout Arkansas, Missouri, and Oklahoma [14]. From 2003 to 2010, NRO decline due to relative crown dieback was estimated at 18% in southeastern forests [15]. One of the key abiotic stressors implicated in oak decline is ozone (O_3_), a compound that is formed by the interaction of sunlight and molecular oxygen and by the interactions of chemicals commonly found in industrial and automobile emissions such as nitrogen oxides and volatile organic compounds. Tree physiology is altered in the presence of O_3_ as evidenced by elevated water use, increased respiration and transpiration, and modified carbon allocation, resulting in decreased tree vegetative growth and life span [16–20]. Forest productivity loss by exposure to O_3_ in the eastern USA has been estimated between 1 and 10% [21]. Ozone stress can further damage NRO indirectly from an increase in disease and insect susceptibility in O_3_–exposed plants [22–24]. Several insect pests are also considered to limit growth and survival of NRO, including red oak borer *Enaphalodes rufulus*, Asiatic oak weevil *Cyrtepistomus castaneus*, carpenter worm *Prionoxystus robiniae*, oak timber worm *Arrhenodes minutus*, and pole borer *Parandra brunnea* [25–27]. Primary damage from these insects also increases tree susceptibility to secondary pests [17, 18, 28].

Due to both the ecological concerns and economic impact from declining forest health, there is a critical need to develop genomic resources and molecular tools that enhance tree improvement and management programs [29]. A number of transcriptome studies on oak species have been leveraged to characterize tree response to biological and environmental stress. The most well studied stress in oak is water stress, with transcriptome studies from seedlings of *Q. lobata*, *Q. suber*, and *Q. robur* that have highlighted alteration of several biological functions including metabolic pathways; energy, lipid, and carbohydrate metabolisms; secondary metabolic, amino acid metabolic, and catabolic processes; sugar transport; photosynthesis; transcription factors; signal transduction; chaperone activity; and pathogenesis-related protein productions [30–32]. Other stress studies from mature oak trees included heat, cold, salinity, oxidative stress, nematode interaction, and fungal pathogenesis that have detected a similarly wide range of differentially expressed primary and secondary pathways [31–38].

Despite the importance of O_3_ in oak decline, there is no information on transcriptome changes in response to ozone. To fill this gap in knowledge, a transcriptome study was designed to assess gene expression differences in NRO induced by ozone exposure. In the forests of Pennsylvania, hourly ambient concentrations of O_3_ typically range between 30 and 80 ppb [39], with occasional occurrences greater than 100 ppb [40]. Four ozone levels were selected for testing. Less than 10 ppb of ozone (little or no ozone after carbon filtration of ambient air) was used as a control, with 80 ppb and 125 ppb as treatments to mimic observed ambient levels. These levels also relate to the U.S. Environmental Protection Agency’s National Ambient Air Quality Standards (NAAQS) for ground-level ozone limits for public health and welfare, which have decreased from 1-h maximum detected levels up to 120 ppb before 1997, to 80 ppb between 1997 and 2015, and to 70 ppb since 2015 (EPA, 2015). A high stress treatment level of 225 ppb was selected as an extreme condition. This is higher than most in situ observations, but close to the 300 ppb level that has often been used in previous reports on ozone-stress studies to produce a strong, reproducible physiological response in model plants [41–43]. By investigating O_3_ stress involved in oak decline, unique molecular-level stress responses by NRO can be determined. Finally, de novo assembly of the RNA sequence data followed by functional annotation of the differentially expressed transcripts was conducted to build a catalog of transcripts in response to O_3_ stress for NRO.

## Results

### Transcriptome sequencing output, de novo assembly and transcriptome quality

More than 334 million raw reads were generated, including 639 Mb from the 454 platform, 2.5Gb from the Illumina MiSeq platform, 23.1Gb from the Illumina Hiseq 2000 platform and 42.3Gb from the Illumina HiSeq 2500 platform. RNA libraries were sequenced from a wide variety of NRO tissues to provide good coverage of the gene space (334,073,559 reads) (Additional file 1: Table S1). To produce a high-quality reference transcriptome, only the longer reads (originating from 454 and Illumina MiSeq) were used for assembly while the data generated from the HiSeq 2500 platform were used exclusively for differential gene expression analysis.

After trimming low-quality bases, adapter removal, transcriptome assembly, and removal of redundant sequences, 52,662 putative transcripts with an average length of 778 bp and N50 length of 1244 bp were generated (Additional file 2: Fig. S1). Transdecoder predicted an open reading frame (ORF) in 38,610 (73%) of the putative transcripts. In order to verify completeness of the transcriptome assembly, putative transcripts were >compared with the Embryophyta database of orthologs (*n* = 1440) by BUSCO; 988 (68.6%) of the single-copy orthologs have a complete match within the oak transcriptome sequences. Another 166 (11.5%) of the single-copy orthologs were found as fragments, and 286 (19.9%) were missing from the oak transcriptome assembly.

While no reference genome is available for *Q. rubra* nor any other species from the red oak clade (subgenus *Quercus* sect. *Lobatae*) [44], three reference genomes from oak species in other clades are available: *Q. lobata* (*Quercus* sect. *Quercus*) [45], *Q. robur* (*Quercus* sect. *Quercus*) [46], and *Q. suber* (*Cerris* sect. *Cerris*) [47]. To assess sequence divergence between this NRO assembly and gene models from the reference genomes, read mapping through Conditional Reciprocal Best BLAST was performed. The proportion of NRO putative transcripts with a match to a gene model in the three oak species genomes was 68.2% to *Q. lobata*, 82.4% to *Q. robur*, and 66% to *Q. suber*, revealing no clear pattern of gene conservation associated with taxonomic relationship. It will be interesting for subsequent phylogenomics studies to determine if the variation in NRO putative transcripts mapping frequency among the species is different among sections of the genus *Quercus* reflects evolutionary distances versus quality and completeness of gene annotations among reference genomes.

### Sequence annotation

Homology-based functional assignments were obtained for a total of 37,535 and 37,880 putative transcripts from NCBI and IPS databases, respectively. Integration of results from both databases yielded annotation for 42,703 (81%) of the putative transcripts. The most common protein matches from NCBI BLAST originated from other woody plant species: *Juglans regia*, *Ziziphus jujuba*, *Theobroma cacao, Prunus persica,* and *Vitis vinifera*. Although an E-value cut-off of 1e-5 was used for the BLAST alignments, the majority of the sequence hits were strongly supported by much lower E-values (Additional file 2: Figure S1). Gene Ontology (GO) terms were assigned to a total of 29,528 (69.1%) annotated putative transcripts. To give a broad overview of annotations, GO term assignments were re-mapped to second-tier GO terms, yielding 70 total terms (Additional file 3: Figure S2), which included: 21,623 putative transcripts that were assigned to terms in the biological process ontology (BP), 20,073 putative transcripts that were assigned to terms in the cellular component ontology (CC), and 24,819 putative transcripts that were assigned to terms in the molecular function (MF) group. The most abundant GO terms for each category were classified as metabolic processes (16,696) and cellular processes (16,125) for BP, cell (14,036) and cell part (13,972) for CC, and binding (16,103) and catalytic activity (15,065) for MF categories. Based on the full set of retrieved GO terms, a total of 10,026 Enzyme Commission (EC) numbers were assigned to annotated putative transcripts, which were utilized to obtain Kyoto Encyclopedia of Genes and Genomes (KEGG) pathway assignments. Categories of retrieved EC numbers included hydrolases (3766), transferases (3267), oxidoreductases (1928), lyases (424), isomerases (346), and ligases (295).

### Analysis of DEGs

The high depth RNA sequencing data was used to profile alterations in gene expression caused by O_3_ stress. Significant DEGs between treatment and control tissue samples were defined at an adjusted *p*-value cut-off of 0.05 and |log2 (fold change)| > 1.

Two-year-old NRO seedlings were exposed to four levels of O_3_ (control, 80 ppb, 125 ppb, 225 ppb), and leaf tissue samples from four biological replicates were taken at three time points (7 h, 14d, 28d). The leaves at the control and 80 ppb levels appeared similar, with no visual injury. Injury was noted at the 125 and 225 ppb exposures. Leaves had the dark red interveinal stippling that is characteristic of moderate O_3_ damage of hardwoods (Additional file 4: Figure S3).

**Fig. 1:**
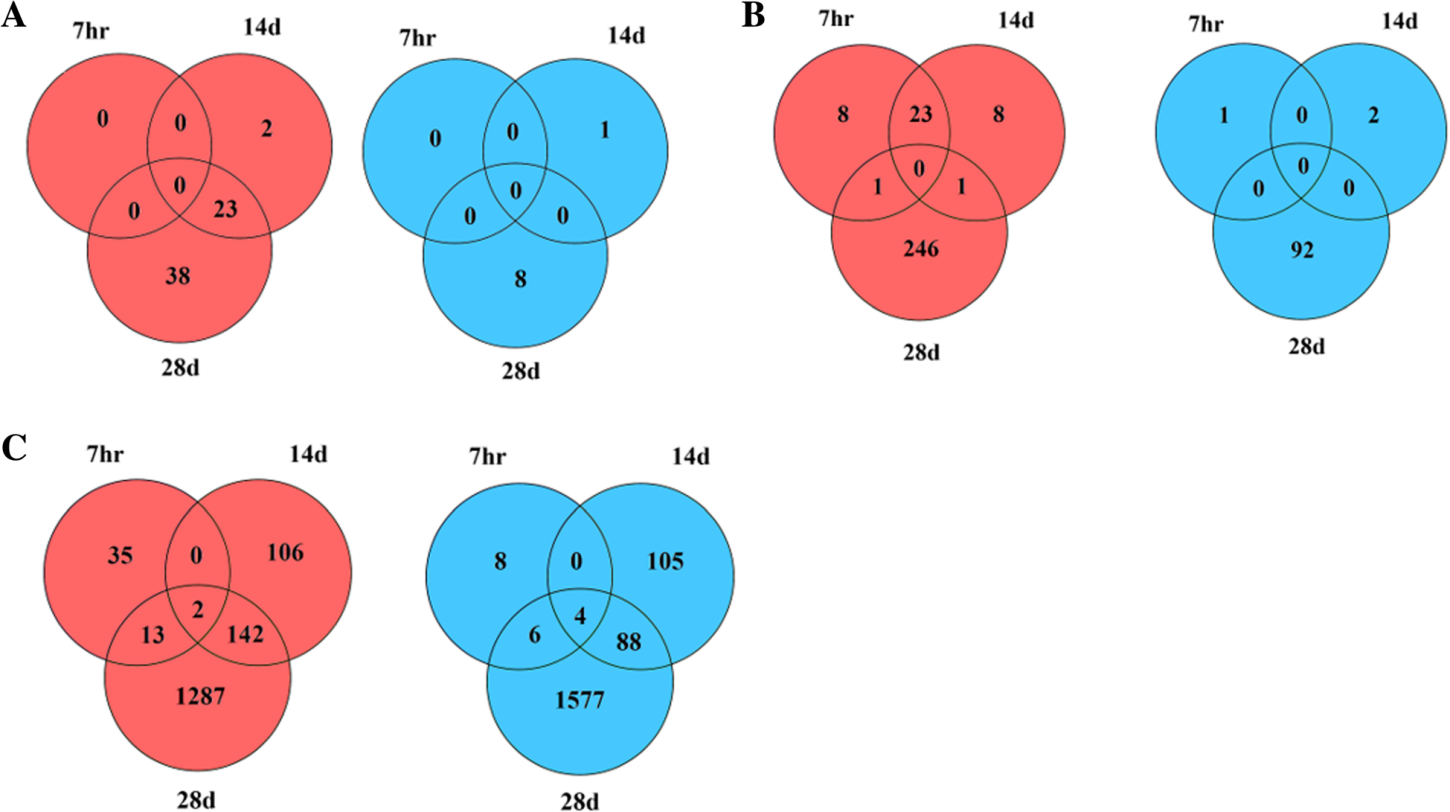
Venn diagrams showing number of DEGs from two-year-old seedlings exposed to O_3_ treatments over time. Times of sampling (7 h, 14 days, and 28 days) are represented by 7 h, 14d, and 28d, respectively. Up- (red) or down-regulation (blue) patterns are also shown for O_3_ concentrations: A) 80 ppb, B) 125 ppb, and C) 225 ppb

Across all elevated O_3_ treatment levels, 4136 DEGs were detected with 2142 transcripts upregulated and 1994 downregulated (Table 1). The number of DEGs identified varied from none found at 7 h (hr) of 80 ppb O_3_, to a maximum of 3120 DEGs after 28 days of 225 ppb O_3_ exposure (Additional file 5: Table S2). The number of DEGs increased both with greater levels of O_3_ and with longer exposure times. The majority of DEGs were found to be unique to each time point. However, a few DEGs were shared among multiple analyses or time points (Fig. 1). DEGs for each O_3_ concentration regardless of time were determined via comparison of O_3_-treated and control samples across all time points using filtering options stated above. While at 80 ppb no DEGs was detected, a total of 33 (32 up-, 1 downregulated) and 70 (52 up-, 18 downregulated) DEGs were identified at 125 ppb and 225 ppb, respectively (Additional file 5: Table S2).

**Table 1:** Number of significant DEGs in response to O_3_ treatment over time

Time	7 h		14d		28d	
Treatment	Up regulated^a^	Down regulated^a^	Up regulated	Down regulated	Up regulated	Down regulated
80 ppb	0	0	25	1	61	8
125 ppb	32	1	32	2	248	92
225 ppb	50	18	250	197	1444	1675

^a^ DEGs regulation patterns and their respective total counts

### GO enrichment categories among DEGs

**Fig. 2:**
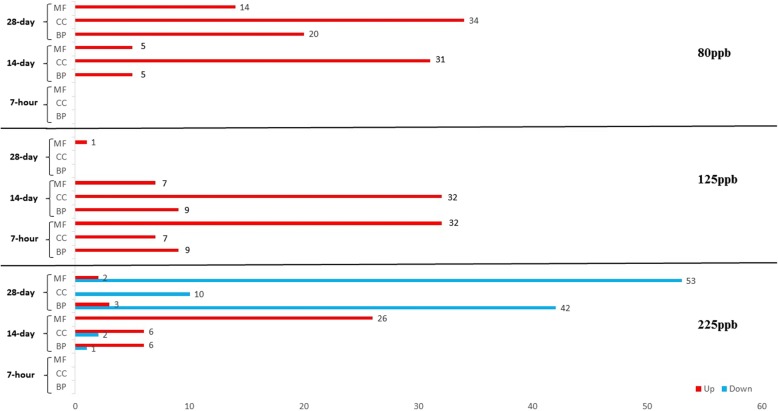
Number of enriched GO terms in unique DEGs of O_3_ treatments over time

**Fig. 3:**
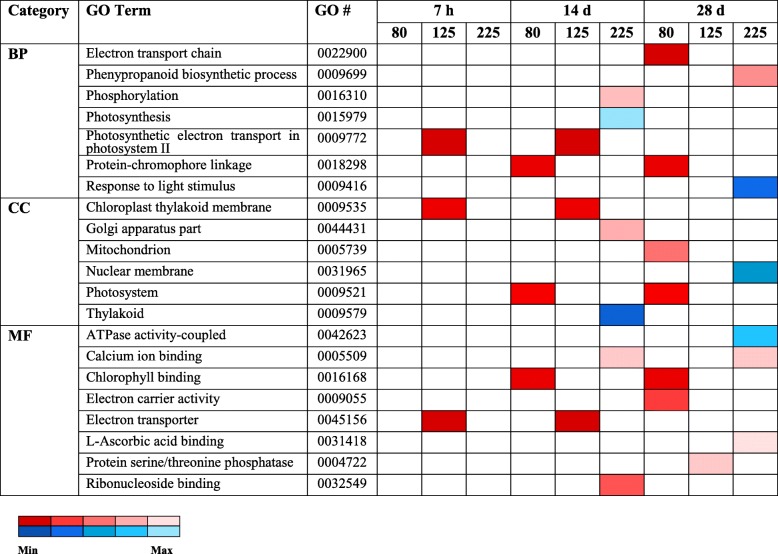
The most highly enriched GO terms in individual O_3_ treatments across time with respect to expression patterns. The expression patterns for up- and down-regulated DEGs are shown in red and blue, respectively. Gradient color represents significance by FDR-adjusted *p*-values. White boxes mean absence of related category in the treatment. Bp: biological process; cc: cellular component; mf: molecular function

GO term enrichment analysis was conducted separately for each treatment to characterize biological functions represented in DEGs. For downregulated DEGs in O_3_ experiments, significantly enriched GO terms were found only at the treatment level of 225 ppb. Enriched GO terms from up-regulated DEGs were identified across all three O_3_ treatments (Figs. 2 and 3). Most down-regulated DEGs, 10 in total, are involved in photosynthesis, and several significant up-regulated DEGs were related to alterations in respiration and photosynthesis (Additional file 6: Figure S4). As photosynthesis activities were found for both upregulated and downregulated genes, we examined the specific genes more closely. For upregulated genes in photosynthesis (at 125 ppb), the genes included two isoforms of photosystem II cytochrome b559 and one gene related to chloroplastic ATP synthase CF0, which both relate to transmembrane activities. In contrast, the downregulated genes at 225 ppb are involved in core chloroplastic activities and organelles (chlorophyll, light receptor, thylakoid lumen, and degradation of damaged proteins in the chloroplast). These genes had specific functional annotations of chlorophyll a-b binding, photosystem I reaction center, photosystem II core complex, LOW PSII ACCUMULATION, psbP domain-containing, and protease Do-like chloroplastic. For the O_3_ concentration-specific DEGs determined regardless of timepoint, enrichment analysis of GO terms for upregulated and downregulated DEGs at 125 ppb were not significant. However, top enriched biological terms for upregulated and downregulated DEGs at 225 ppb were cysteine metabolism and steroid metabolism, respectively (Additional file 6: Figure S4).

Regulation patterns of GO terms are shown. Bp: biological process; cc: cellular component; mf: molecular function.

### KEGG pathway enrichment analysis of DEGs

**Fig. 4:**
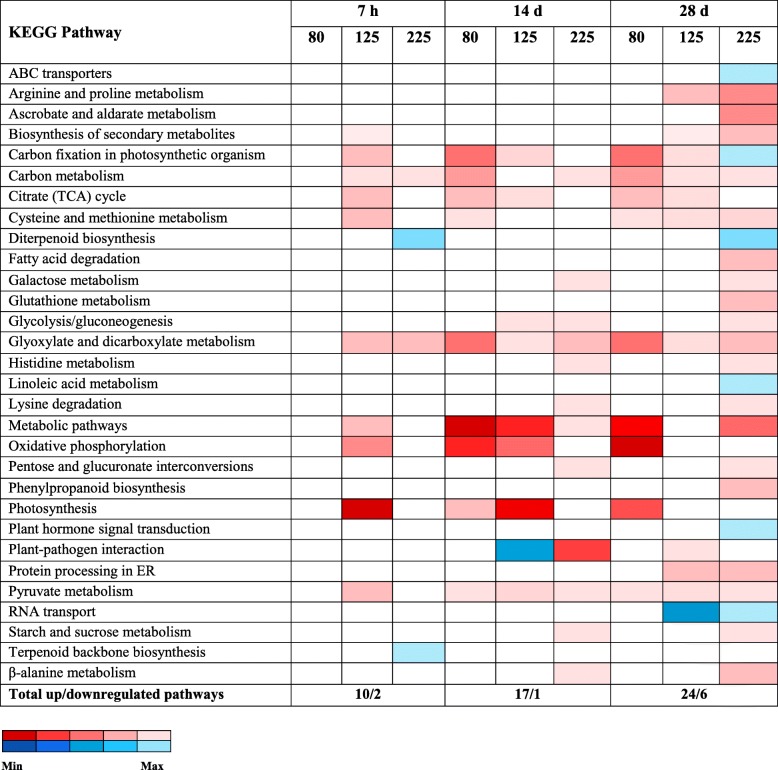
Enriched KEGG Pathways of DEGs with respect to their expression patterns in individual O_3_ treatments across time. The expression patterns for up- and down-regulated DEGs are shown in red and blue, respectively. Gradient of color represents FDR-adjusted *p*-value for respective regulation patterns (up/down). White boxes mean absence of statistical significance for the related pathways due to the treatment

KEGG pathway enrichment tests were conducted with the upregulated and downregulated DEGs identified in the GO enrichment analysis (above). The number of perturbed pathways illustrated an impressive diversity of biochemical functions, which increased in scope both with time of exposure and O_3_ concentration (Fig. 4; Additional file 7: Table S3). The three most highly enriched upregulated KEGG pathways were oxidative phosphorylation, metabolic pathways, and photosynthesis, while the most downregulated KEGG pathways were plant-pathogen interactions, RNA transport, and diterpenoid biosynthesis. For the O_3_ concentration-specific DEGs, KEGG pathways analysis of upregulated DEGs at 125 ppb detected photosynthesis as the top enriched biological pathway (Additional file 7: Table S3) with involvement of three DEGs, however, downregulated DEGs were not enriched for photosynthesis activities. Enrichment analysis of upregulated DEGs at 225 ppb detected top significant KEGG pathways as sulfur metabolism (Additional file 7: Table S3), while downregulated DEGs were not significant.

### Time-series analysis of DEGs

**Fig. 5:**
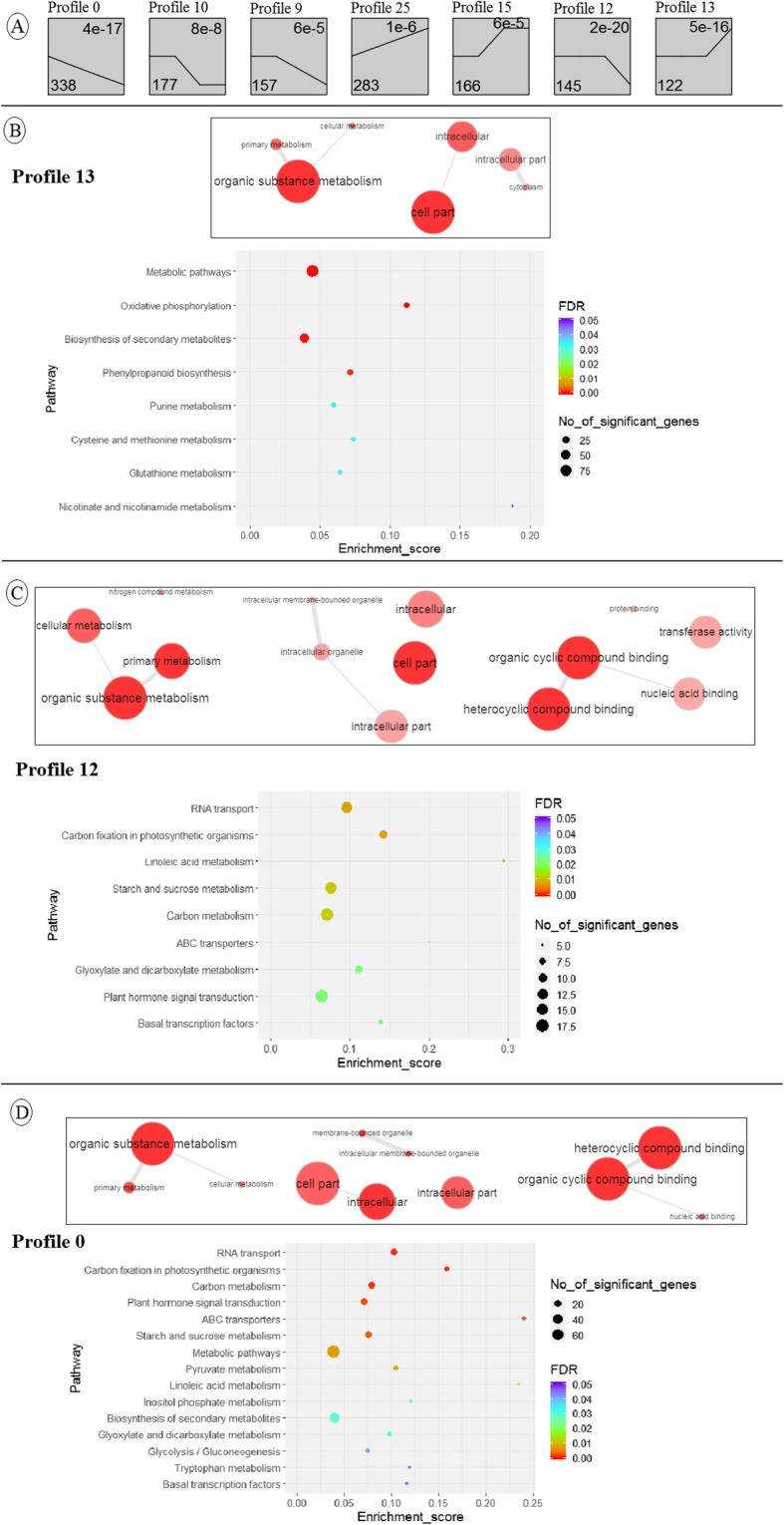
Time-series and enrichment analysis of DEGs associated with ozone-exposed samples versus their control. A) Overall temporal expression profiles of DEGs with statistically significant clusters. On top of each box, profile number is represented. Left to right of X-axis in each profile represents over time pattern. Top right of individual profile is the profile enrichment *p-value*, and lower left is the number of DEGs assigned to each model profile. B-D) Enriched GO terms and KEGG pathways of DEGs in profile numbers 13, 12, and 0. For the GO terms, the larger the size of circle, the higher the frequency; and darker the red color, lower the *p-value*. For the KEGG pathways, enrichment score is the number of significant genes divided by background genes of respective pathway; FDR is the false discovery rate corrected *p-value*

Characterization of temporal dynamics of DEGs following O_3_ induction using Short Time-series Expression Miner software (STEM) software [48] was performed by clustering DEGs based on the similarity of their temporal expression patterns. STEM analysis clustered 1388 DEGs in seven significant profiles, of which most DEGs grouped in the profiles representing downregulation pattern over time (Fig. 5a; Additional file 8: Table S4). Functional annotation of DEGs associated with significant clusters detected enriched GO terms and KEGG pathways only in profiles 0, 12, and 13. For DEGs associated to profile 13 with the upregulation pattern over time, the top two significant biological functions were cell part and metabolic pathways (Fig. 5b). The top two enriched biological pathways of clustered DEGs in both profiles of 0 and 12 with the downregulation pattern over time were organic substance metabolism and RNA transport (Fig. 5c-d).

### Detection of co-expressed genes upon ozone stress

**Fig. 6:**
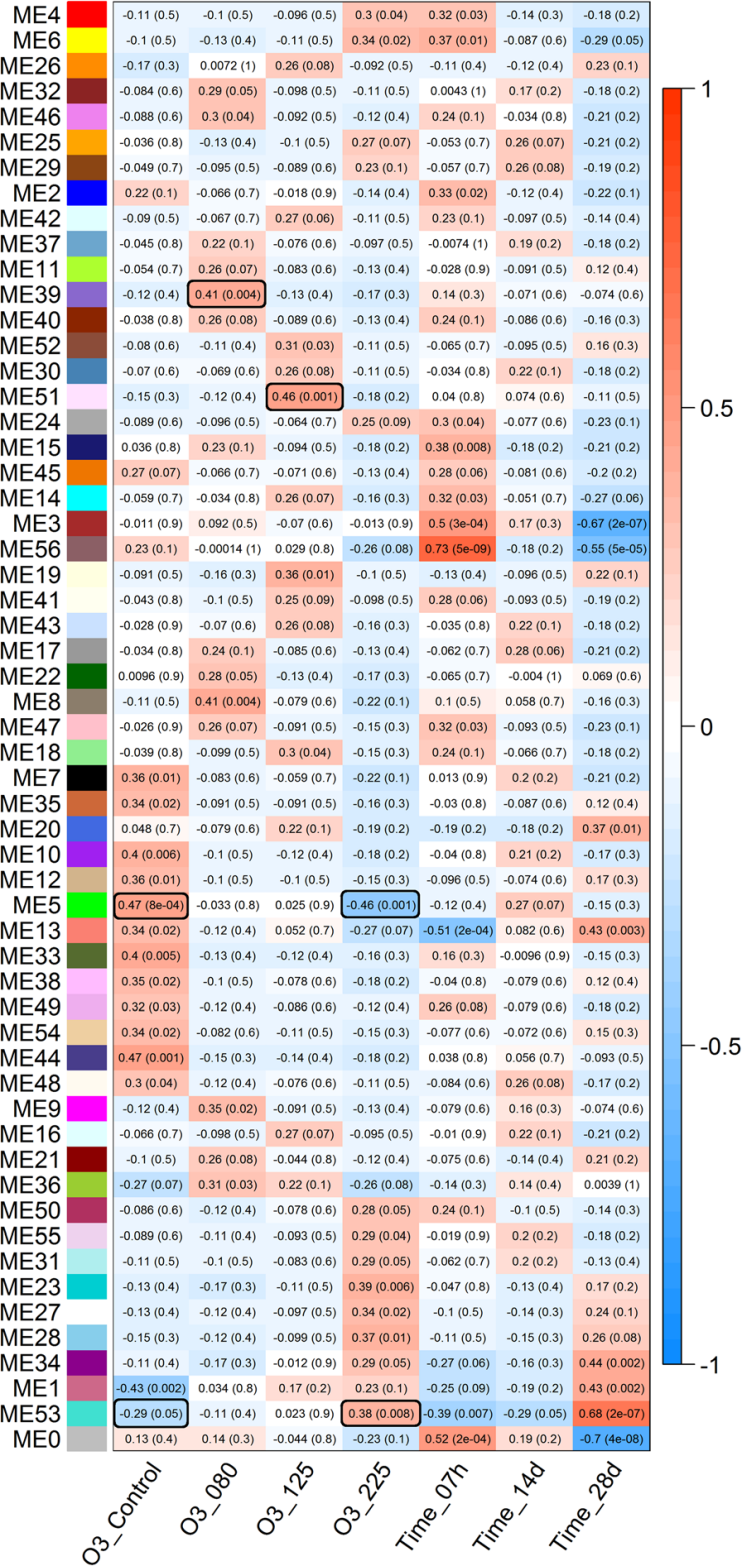
Module-factor relationship summarizing co-expressed gene clusters in respective module eigengene (ME) in northern red oak in response to ozone exposure. Individual ME with respective color is indicated on the Y axis, and ozone treatments and exposure time points are shown on the X axis. In each box correlation coefficient and its *p-value* in parenthesis indicating correlation significance of respective treatment/time per detected ME. The color gradient shows expression profile of respective treatment/time in each ME. Highly correlated modules responding to ozone concentrations of 80, 125, and 225 plus differential expression profile of 225 ppb versus control are highlighted in black

To identify co-regulation of gene clusters during ozone treatments, weighted correlation network analysis (WGCNA) was carried out using all samples. The total of 44,078 genes were clustered in 57 modules (Fig. 6), with a range of 121 (ME56) to 12,492 (ME0) genes per module. The modules represent subsets of genes with highly correlated patterns of expression. For each module, a module eigengene (ME) has been calculated to represent the first principal component of the module. The eigengene can be interpreted as an “average” expression value representing all the genes in the module. Module-factor relationships were calculated to assess correlation of gene clusters to experimental factors. This provides a p-value indicating how well modules are correlated with each factor in the experiment. ME39 with 260 genes was the most correlated cluster responding to 80 ppb of O_3_. The most significant biological KEGG pathways enriched in ME39 were sesquiterpenoid and triterpenoid biosynthesis, pyruvate metabolism, and biosynthesis of secondary metabolites (Additional file 9: Table S5). ME51 was the most correlated module responding to 125 ppb of O_3_. It contained 187 genes, of which the most represented biological functions were protein processing in endoplasmic reticulum, defense response, and response to stimulus (Additional file 9: Table S5). ME5 was the most correlated module of genes responding to 225 ppb of O_3_ that comprised of 1039 genes, of which the most significant biological KEGG pathways were metabolic pathways, carbon metabolism, and biosynthesis of secondary metabolites (Additional file 9: Table S5). Factor comparison in the co-expression module-factor relationship (Fig. 6) indicated that two modules, ME5 and ME53, were differentially co-expressed in response to 225 ppb of O_3_ (versus control). Aside from ME5 described above, ME53 contained 6248 genes with the most significant KEGG pathways involved in spliceosome, metabolic pathways, and protein processing in endoplasmic reticulum (Additional file 9: Table S5).

### Significant enriched DEGs in plant-pathogen interaction pathway

While KEGG pathway mapping and GO term enrichment analysis are powerful methods to determine the overall biological and metabolic processes for a set of genes, both analyses are limited by the number of genes that have been accurately annotated. With de novo assembled transcriptomes and sequence similarity-based functional annotation, direct examination of the gene lists can reveal additional important pathways. In O_3_-exposed samples, a total of 14 upregulated and one downregulated stress response DEGs were found that also had an annotation to the plant-pathogen interaction pathway from KEGG (Table 2).

**Table 2:** DEGs involved in plant-pathogen interaction pathway

Ozone treatments with respective regulation pattern	Putative transcripts id	ME^b^	Annotation	|LogFC|^a^	KEGG entry	Length (bp)
125 ppb at 14 day-down	comp19736_c0_seq3	ME0	Disease resistance RPM1	6.26	K13457	682
125 ppb at 28 day-up	comp21410_c0_seq1	ME1	Heat shock cognate 80	1.43	K04079	1281
	comp31660_c0_seq2	ME1	Enhanced disease susceptibility 1	2.43	K18875	2272
	comp31660_c0_seq1	ME1	Enhanced disease susceptibility 1-like	2.55	K18875	946
	comp26463_c0_seq2	ME1	Calcium-dependent kinase 10	2.46	K13412	2130
	comp28434_c0_seq1	ME53	Calcium-binding CML23	2.03	K13448	661
	comp18561_c0_seq1	ME1	Lipase_3 domain-containing	3.52	K18875	367
	comp94592_c0_seq1	ME53	Calcium-binding CML45	3.27	K13448	786
225 ppb at 14 day-up	comp31273_c0_seq7	ME51	Disease resistance At1g12280 (LRR and NB-ARC domains)	3.61	K13459	845
	comp18481_c0_seq1	ME53	Calcium-dependent kinase	5.63	K13412	596
	comp9320_c0_seq1	ME0	26-like	3.56	K13412	833
	comp21573_c0_seq1	ME0	Calcium-dependent kinase 26-like	1.43	K04079	888
	comp31660_c0_seq5	ME5	Heat shock cognate 80-like	1.49	K18875	1932
	comp23142_c0_seq3	ME31	Enhanced disease susceptibility	1.25	K20536	1335
	comp31028_c0_seq1	ME5	Mitogen-activated kinase 3 Respiratory burst oxidase homolog A	2.04	K13447	3505

^a^ Variation in logFC indicates the amount of log-based changes of gene expression level in the treatment compared with respective control sample

^b^ ME refers to the module number from the gene coexpression network analysis

### Identification of DEGs involved in terpenoid biosynthesis pathway

The DEGs induced in O_3_ stress were involved in several pathways related to terpenoids, including biosynthesis of secondary metabolites, terpenoid backbones, and diterpenoids. Ozone stress resulted in three terpenoid biosynthesis related DEGs (Table 3). The number of downregulated DEGs was higher than upregulated DEGs. Perturbed genes covered a set of enzymatic activities including synthesis, oxidation, and reduction.

**Table 3:** DEGs involved in terpenoid biosynthesis pathway

Treatments that induced differential expression	Putative transcripts id	ME^a^	Annotation	Role in terpenoid pathway	|LogFC|	KEGG entry	Length (bp)
Shared 7 h&28d at 225 ppb, Downregulated	comp29400_c1_seq4	ME53	β-Amyrin 11-oxidase-like	Diterpenoid	1.83	K04123	289
225 ppb at 7 h-Downregulated	comp28709_c0_seq1	ME2	3-Hydroxy-3-methylglutaryl-coenzyme A reductase	Terpenoid backbone	1.99	K00021	1630
225 ppb at 28 day-Upregulated	comp313540_c0_seq1	ME53	Geranylgeranyl pyrophosphate synthase	Terpenoid backbone	5.99	K13789	227

^a^ ME refers to the module number from the gene coexpression network analysis

## Discussion

Although several transcriptome studies have previously identified candidate genes and pathways involved in response to multiple biotic and abiotic stressors in various oak species [30–32, 35, 36], knowledge at the genomic level of the effect of increased ground-level O_3_ toxicity to NRO is lacking. In this transcriptome study, NRO leaf tissues were exposed to four levels of O_3_ treatments in a time-series (7 h, 14 day, 28 day) experiment, in order to reveal candidate genes and gene products key to NRO response to this abiotic stress.

### Transcriptome assembly and annotation of putative transcripts

The de novo transcriptome assembly generated a total of 52,662 putative transcripts as a resource to further genomic research in NRO and related oak species. The total average length and N50 contig length are comparable to the reference transcriptomes developed to date for other forest trees [49–52]. More than 80% of the NRO putative transcripts could be functionally annotated, and the GO term assignments indicated that a broad set of fundamental metabolic processes and biological pathways were included. This distribution of GO terms is consistent in scope with previous reference de novo transcriptome studies, including oak [30, 36, 37] and non-oak species [53–55]. Thus, the transcriptome reported here provides a good reference for NRO studies. However, further improvements in gene space coverage and structural and functional annotations could be achieved through assembly of a reference genome, complete with full length gene models, for *Q. rubra*.

### Impacts of ozone exposure to NRO leaves among ozone concentrations, time-specific ozone concentrations, and time-series exposures

In this study, gene expression patterns in the NRO seedlings varied by both time and concentration of O_3_ stress treatments. At the lowest treatment level of 80 ppb, gene expression did not differ from the control at the 7 h time point. In contrast, at higher O_3_ concentrations, gene expression was actively responding to the treatments even at first time point of 7 h. Overall, the number of differentially expressed genes increased as both a function of time and increasing O_3_ levels.

**Fig. 7:**
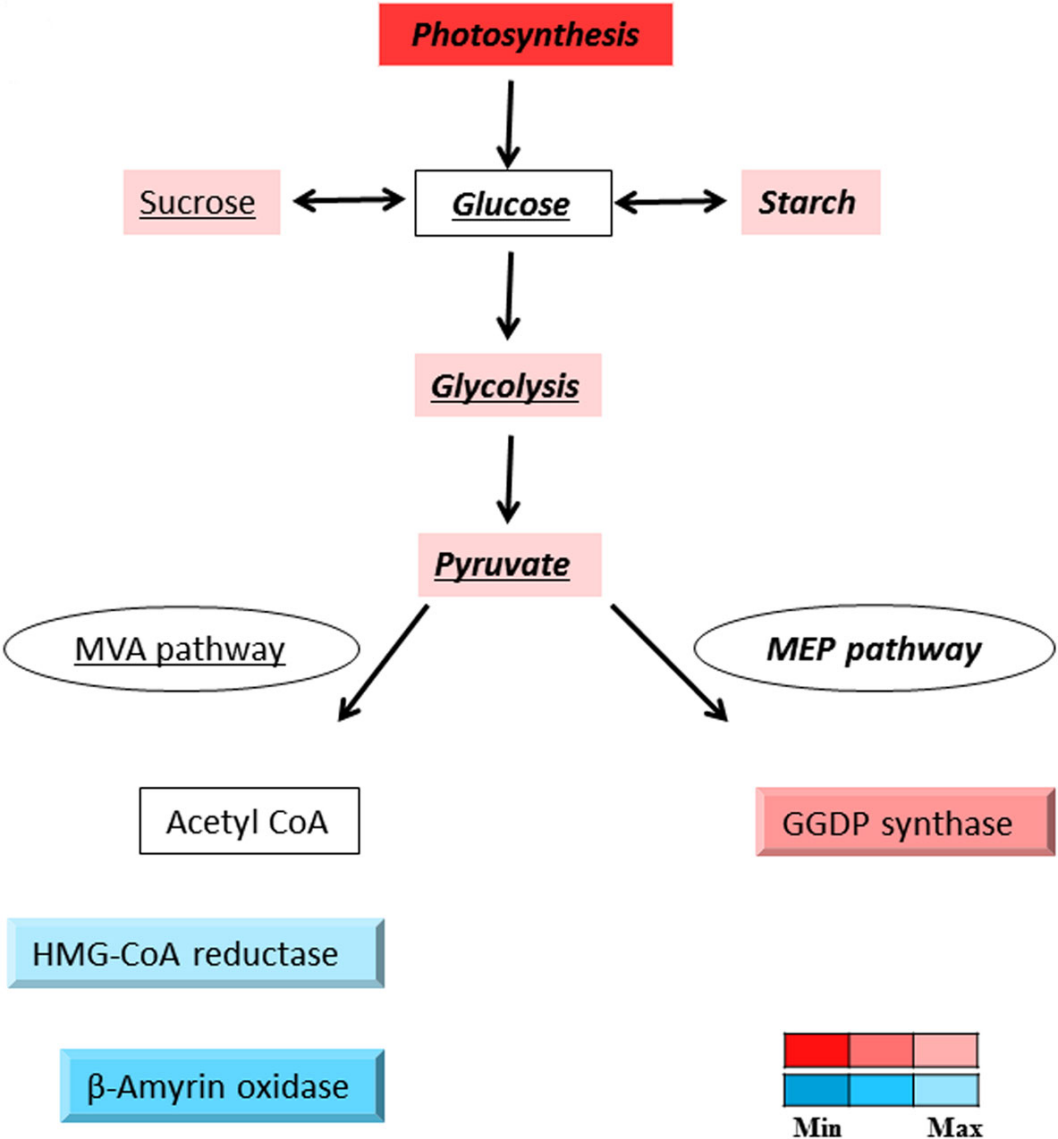
Overall impact of ozone on terpenoid biosynthesis in northern red oak leaves. Leaves from seedlings exposed to ozone (mean FDR-adjusted *p*-values values of all ozone concentrations and exposure times); 3-Hydroxy-3-methylglutaryl-coenzyme A (HMG-CoA) reductase is an ATP-dependent enzyme, necessary for biosynthesis of mevalonic acid, a key compound in isopentenyl diphosphate (IPP) formation. β-amyrin 11 oxidase, an essential cytochrome P450 enzyme, forms different terpenoid compounds through oxidation and glycosylation of β-amyrin. Geranylgeranyl pyrophosphate (GGDP) synthase adds IPP units to terpenoid skeleton to biosynthesize diverse types of terpenoids including mono-, di-, tri- and tetraterpenoids. Significance of expression patterns (FDR-adjusted p-value) are represented by color gradient, with upregulation and downregulation in red and blue colors, respectively. Bold and italic processes occur in plastids; underlined processes occur in cytosol; bold, italic, and underlined processes occur in either plastid or cytosol. MVA: mevalonic acid; MEP: methylerythritol phosphate

Perturbation of carbon metabolism genes was observed among 125 and 225 ppb O_3_-exposed tissues, as well as temporal expression pattern analysis. In addition, altered metabolic pathways during the short-term exposure (7 h) at the two higher O_3_ levels of 125 ppb and 225 ppb and time-series analysis were carbohydrate, amino acid, terpenoid biosynthesis and energy production. Genes involved in these biological pathways were also co-expressed in response to O_3_ as they were assigned to co-expression modules, ME5 and ME53. Biological pathways have also been perturbed in the seedlings of *Q. lobata* upon drought stress [32] and seedlings of *Q. suber* during ectomycorrhizal interaction [56]. Higher expression levels of genes participating in the glycolysis and citrate (TCA) cycles during O_3_ exposure could be expected to result in increased ATP synthesis, as documented previously in multiple plant species [57–59]. Consistent with previous research [60–62], energy production and carbohydrate fixation pathway gene activities were also affected in our study. In the long term, however, increased carbon use can lead to damaged photosynthetic machinery, a phenomenon that ultimately results in reduced ecological and economic productivity [63, 64], as evidenced by early leaf senescence in trees due to ozone stress in nature [65, 66]. Biosynthesis of several defensive secondary metabolites including terpenoids is modulated in plants in response to environmental changes, pathogens and herbivores [67–69] as well as oaks in response to environmental changes and soil-borne microbes [32, 56]. Terpenoids are a class of bioactive compounds with antimicrobial, anti-herbivore, and insecticidal functions, which can be involved in attenuation and suppression of O_3_-induced oxidative-stress damages [70–72]. Five different types of terpenoids, mono-, di-, tri-, tetra-, and sesquiterpenoid, are biosynthesized through sequential condensation of isoprene unit blocks resulting from cytosolic mevalonic acid (MVA) or plastidal methylerythritol phosphate (MEP) pathways. Sesqui- and triterpenoids are produced through the MVA pathway, whereas mono-, di- and tetraterpenoids are biosynthesized through the MEP pathway [55, 67]. In this study, perturbation of terpenoid biosynthesis due to O_3_ exposure was a result of changes in expression levels of three enzymes; enzymes involved in the MVA pathway were downregulated while those in the MEP were upregulated (Table 3; Fig. 7). Modulation of these pathways upon O_3_ exposure in NRO is consistent with reports for oxidative stress studies in other woody plants [71, 73].

The stress treatments of NRO seedlings for medium length of O_3_ exposure (14d) resulted in alterations in GO terms that predict alterations in protein levels of exposed plants at all O_3_ levels; co-expressed genes were clustered in modules ME5, ME51, and ME53. These terms included protein complex, protein-chromophore linkage, cysteine and methionine metabolism, histidine metabolism, and lysine degradation. Furthermore, overexpression of sulfur metabolism genes at 225 ppb O_3_ exposure, and cysteine and methionine metabolism in either over-time analysis or O_3_ exposure of 225 ppb was observed. Modulation of amino acid metabolism upon exposure of oak seedlings to water stress [32] and ectomycorrhizal contact [56] might imply this pathway as a common stress responsive mechanism during exposure to abiotic stimuli, which is in agreement with results of previous studies related to ozone-exposed plants [60, 62]. In plants, reactive oxygen species (ROS) can react with thiol and sulfur-containing groups of cysteine and methionine [74] and lead to conformational changes in histidine and lysine amino acids, which impairs protein function and increases susceptibility to proteolytic reactions [75]. Furthermore, ROS trigger protein oxidation, a phenomenon which often causes irreversible covalent alteration of the protein structure [74]. The expression of plant-pathogen interaction pathway and related genes are reported to be altered in response to biotic and abiotic stimuli in plants [76, 77] such as *Q. robur* seedlings exposed to waterlogging [31]. Furthermore, activation of defense pathways can lead to priming of unexposed tissues for faster gene expression responses to stress and may lead to defense reactions such as hypersensitive response (HR). In our study, co-expression of plant-pathogen interaction pathway/defense response was observed in all O_3_ treatments, where these defensive responses were assigned to modules ME5, ME39, ME51, and ME53. Among these pathways and responses, upregulation of an “enhanced disease susceptibility” gene and downregulation of “disease resistance RPM1” gene could potentially alter HR and programmed cell death, which would ultimately result in cell vulnerability and impairment. In this study, induction of calcium-dependent putative transcripts, may indicate increased levels of defense signal-transduction systemically to distal plant tissues [78]. The amino acid glutamate plays a key role in long distance signaling, priming defense responses through systemic acquired resistance pathways [78]. In past studies, exposure to O_3_ was reported to result in upregulation of plant-pathogen interaction pathways such as pathogenesis-related proteins 1–4 and small heat shock proteins; our study differed from previous reports in that pathogenesis-related proteins were not differentially expressed in the NRO seedlings [79–81].

Photosynthesis and ATP production pathway genes were observed to be consistently upregulated after long term (28d) exposure to O_3_ and among 125 ppb-exposed tissues, as it has been documented in several studies [60–62, 82]. However, evidence of altered carbon fixation through 1,5-bisphosphate carboxylase (Rubisco) gene expression was inconsistent among exposure times and O_3_ levels. However, co-expression analysis showed that photosynthesis- and ATP production-related genes were both clustered in the modules ME5 and ME53. In addition to latter modules, ME39 and ME51 also contained ATP production-associated genes. After long term O_3_ exposure (28d), downregulation of Rubisco at the highest O_3_ concentration was observed. Several explanations for downregulation of Rubisco have been proposed including inhibited transcription, mRNA degradation, and reduction of stomatal conductance in response to O_3_ [83, 84]. Modulation of stomatal conductance alters the uptake of atmospheric CO_2_ to intercellular spaces, which ultimately affects carbon fixation and sugar deposition [85]. The indication of decreased carbon fixation from altered gene expression patterns in the treated NRO seedlings is consistent with previous studies related to oaks and other woody trees [85–87]. In our study, differential gene expression results suggested that photosynthesis was upregulated, rather than being suppressed. Although photosynthesis is reported to be decreased during elevated O_3_ in some plant systems [88–91] and oak species (*Q. lobata* and *Q. suber*) exposed to drought [30, 32], it is typically increased in younger tissues in response to stress [92–94]. However, photosynthetic rates of tree seedlings have been reported to be less sensitive to O_3_ than mature trees [92–94]. For plants to recover from damage to photosynthetic compartments, seedlings need to assimilate the sugar and starch that are essential for growth. This is generally accomplished through carbon shifts allocation to the roots. However, O_3_ and other photosynthetic poisons can alter shifts carbon in favor of the shoot, which along with increased photosynthetic rates can result in early leaf senescence and decreased seedling growth [95]. In our study, after 28 days of exposure to high O_3_ levels, many genes associated with the plant defense cascades were upregulated. For example, ROS can perturb the plant-pathogen interaction pathway, which in turn activates HR through either effector-trigger immunity (ETI) or pathogen-associated molecular pattern-triggered immunity (PTI) that circumvent O_3_ induced damages. Overexpression of two isoforms of “enhanced disease susceptibility 1” (EDS1)”, as well as induction of heat-shock protein (HSP) and calcium-dependent/binding genes in the O_3_-treated NRO seedlings might thus be attributable to stimulation of HR by either ETI or PTI. Higher levels of ROS in cells ultimately leads to programmed cell death [96]. Therefore, consistent with past studies [60, 62], increased expression of HSP and amino acid glutathione, an important anti-oxidant, plus other ROS scavengers in plant tissues, as we observed, may provide detoxification methods that diminish O_3_ induced damages [16, 57, 97].

DEGs that were observed to be upregulated in the O_3_ experiment included transcription factors such as WRKY and other genes known to be involved in host defense responses, including HSP and thaumatin-like protein genes. Heat-stress transcription factors play an important role in regulation of expression of genes such as the HSP protein gene which responds to stresses and promotes plant defense reactions. Thaumatin-like proteins are PR proteins that are induced in response to pathogen/pest attack and are involved in plant resistance responses [98]. In other oak seedling studies, another closely-related HSP family (HSP20) [30, 32] and several transcription factors such as WRKY [30, 32, 56] and those regulating HSPs [56] were differentially expressed during drought and fungal stresses. Modulation of multiple transcription factors including WRKY upon ozone exposure were also documented in several other plants [60, 62]. On the other hand, the observed downregulation of ABC transporter (annotated as ABC transporter family G member 11 [ABCG 11]), LRR receptor-like DEGs and terpenoid pathway genes after ozone exposure of the NRO plants suggests some active defense mechanism may be disturbed by this stress, potentially increasing susceptibility to pathogens and pests. Perhaps such downregulation of gene expression also represents reduction in resource use for tissues already proceeding to apoptosis and senescence. Modulation of ABC transporter and LRR receptor-like genes during O_3_ stress is consistent with oak seedling studies associated with fungal and drought stresses [31, 32, 56]. In *Arabidopsis*, ABCG 11 mutants lose water maintenance and plant defense functionality through disturbance of cuticle membrane lipid transfer [99]. LRR receptor-like genes regulate diverse developmental and defense related processes including non-host-specific defense reactions induced pathogen infection [100].

While the use of four independently sequenced biological replicates in this study lends statistical confidence to the results, the limited red oak genetic background is a limitation. The genes and pathways reported here need to be further queried, preferably through independent repeats of this experiment using additional red oak genotypes and ozone levels. This could yield information about how well these responses are conserved across red oak populations.

## Conclusion

In this paper we reported the development of a reference transcriptome for NRO developed from deep sequencing, and assembly, of RNAs from wide variety of NRO developmental stages. The reference transcriptome assembly consists of 52,662 unigenes, of which more than 42,000 transcripts were annotated by sequence homology and by gene ontology to a broad array of functional classifications. Over 4100 differentially expressed genes were detected in response to a time course of O_3_ stress at 3 levels, versus untreated controls. Although much has been learned through previous ecological and physiological studies on the effects of ozone-stress in NRO and other forest trees, to the best of our knowledge this is the first study of genome-wide gene expression responses of NRO plants to ozone stress. Exposure to elevated ozone levels led in both cases to activation of a cascade of defense gene expression, including altered carbohydrate, amino acid, lipid, and terpenoid biosynthesis as well as altered photosynthesis and ATP production pathway genes. The ozone toxicity is example of oxidative stresses, during which ROS are produced, impair lipid and protein functions and increase susceptibility to proteolytic reactions. Enhanced glutathione as suggested by upregulated gene expression (temporal and concentration-dependent) in the leaves indicated activation of antioxidant-detoxification pathways in response to the oxidative stresses imparted by ozone treatments. Prolonged exposure of oak trees to this external stimulus could increase susceptibility to secondary pests and pathogens, contributing to oak population decline. Further characterization of the candidate genes from this study should be pursued as opportunities to enhance resistance against biotic and abiotic stressors through oak breeding and reforestation programs. Additional genomic resources, such as a reference genome for *Q. rubra*, would further support research on NRO adaptation and resistance to different stresses.

## Methods

### Plant materials and ozone treatments

Tissue samples were collected from two adjacent mature NRO trees on the campus of Purdue University, West Lafayette, Indiana (accessions SM1 and SM2) [101]. The tissues sampled included dormant twigs, immature twigs, developing acorns, emerging leaves, catkins, emerging leaf buds, late growth stage (season) damaged leaves, late growth stage undamaged leaves, late growth stage damaged twigs, and late growth stage undamaged twigs. All tissues were flash frozen in liquid nitrogen immediately after collection, and then kept frozen in either in liquid nitrogen or on dry ice during transport to the lab for storage at − 80 °C. These materials were sequenced using MiSeq and 454 instruments and used exclusively for transcriptome assembly.

#### Ozone stress

Two ozone exposure experiments were performed. For the initial experiment, open pollinated acorns collected from SM1 were germinated and grown for two years in the greenhouse under normal ambient conditions. In the summer of 2011, 24 two-year old seedlings were randomly assigned among four continuous stirred tank reactor (CSTR) chambers [cylindrical in shape, with dimensions of 107 cm (diameter) × 122 cm (height)] [102], with six seedlings transferred into each chamber. Each CSTR chamber was equipped with an external overhead light source (400 watt lamps [~ 15 klx]) producing light quality similar to natural sunlight. The seedlings were acclimated to the chambers for 2 weeks at normal ambient growing conditions, after which the O_3_ concentrations were adjusted to a different level in each chamber, at < 10 ppb (control), 150 ppb, 225 ppb, and 300 ppb. The specific ozone levels were accomplished by an air-intake scrubbing system consisting of activated charcoal filtration unit that lowered ambient air ozone levels in the greenhouse to < 10 ppb hourly average. Ozone was then added to each CSRT chamber via a controllable micro-metering system with concentrations monitored with a TECO Model 49 O_3_ analyzer and data logger/computer recording system in each chamber [103]. The augmented O_3_ was delivered in square-wave fashion for 7 day/wk., eight hours a day (0900 h to 1559 h) for 28 days, mimicking diurnal ozone fluctuation. In treatments greater than ambient, cumulative ozone exposure ranged from 864 to 1728 ppb h for 7 h treatments, from 13,992 to 25,152 ppb h for 14 day exposures, and from 28,008 to 50,328 ppb h for 28 day exposures. The metric ppb h was calculated as (ppb × 8 h × # days). During non-fumigation hours, seedlings remained within the chambers with the doors open to the charcoal filtered air and environmental conditions within the greenhouse. Three to four leaves were collected from different areas in the canopy (lower, mid and upper) at each of the three time points (7 h, 14 days, 28 days) from all biological replicates. Leaves were flash frozen in liquid nitrogen immediately after collection, and then kept frozen in either in liquid nitrogen or on dry ice during transport to the lab for storage at − 80 °C. For each replicate, the leaves were pooled prior to RNA extraction. After isolation, equal amounts of RNA from the replicates were pooled by treatment level prior to sequencing by a 454 instrument for use in transcriptome assembly.

A second O_3_ exposure experiment was performed with 48 two-year open-pollinated seedlings grown from acorn collected from accession SM1. In this experiment, four seedlings were used as biological replicates in each of four CSTR chambers, treated at O_3_ concentrations adjusted to: < 10 ppb (control), 80 ppb, 125 ppb, and 225 ppb. Less than 10 ppb of ozone (little or no ozone after carbon filtration of ambient air) was used as a control, with 80 ppb and 125 ppb as treatments to mimic observed ambient levels. These levels also relate to the U.S. Environmental Protection Agency’s NAAQS for ground-level ozone limits for public health and welfare, which have decreased from 1-h maximum detected levels up to 120 ppb before 1997, to 80 ppb between 1997 and 2015, and to 70 ppb since 2015 (EPA, 2015). A high stress treatment level of 225 ppb was selected as an extreme condition. This is higher than most in situ observations, but close to the 300 ppb level that has often been used in previous reports on ozone-stress studies to produce a strong, reproducible physiological response in model plants [41–43]. Leaf samples were collected and tracked individually from each of the biological replicates at three time points (7 h, 14 days, 28 days) for the 4 ozone treatment levels. Leaf samples were collected and processed as described above. RNAs were isolated and replicates sequenced separately on Illumina instruments to generate data for use in differential expression analysis.

### RNA purification, library construction and transcriptome profiling

The frozen tissue samples were powdered by grinding in liquid nitrogen and transferred back to − 80 °C freezer conditions if not immediately extracted for RNA. Total RNA was extracted from the powdered tissue samples following a modified CTAB isolation method [104] with lithium chloride precipitation. RNA quality was assessed by capillary electrophoresis using the Agilent Bioanalyzer 2100 (Agilent technologies).

Libraries for 454 instrument sequencing were constructed as per supplier’s instructions for the Titanium reagents with modifications as described as in [105]. The libraries were sequenced at Pennsylvania State University using an FLX+ 454 DNA sequencer (Roche). For the initial O_3_ experiment, equal amounts of RNA from individual biological replicates were pooled into a single sample for each ozone treatment level. Two additional 454 libraries were constructed from the parent tree samples - one from a pooled set of equal amounts of RNA from above ground tissue samples and one from a pooled set of below ground tissue samples.

For the second O_3_ stress experiment, biological replicates were independently barcoded for sequencing. Illumina TruSeq libraries were prepared for each of the replicate RNA samples, following manufacturer protocols, then sequenced on an Illumina HiSeq 2500 instrument at Pennsylvania State University.

All RNA-Seq data are available in the NCBI Sequence Read Archive database under BioProject accession number PRJNA273270.

### RNA-seq preprocessing, de novo assembly and quality assessment

The quality of generated RNA-Seq data was inspected by FastQC software [106] and low-quality reads (mean Phred score < 20) were cleaned by Trimmomatic using default parameters [107]. Only reads originating from the 454 instrument or the MiSeq instrument were included in the assembly, due to their longer read lengths. Trimmed reads were assembled by Trinity (version downloaded on 2012-10-05) [108]. The assembly was further refined by cd-hit-est v4.6.1 with a sequence identity threshold of 0.95 to collapse isoforms and reduce assembly redundancy [109].

All transcript names begin with “Quercus_rubra_120313_” to indicate transcriptome origin and version. This part of the transcript name has been removed from the text for brevity. For example, transcript “Quercus_rubra_120313_comp102049_c0_seq1” is referred to in the text as “comp102049_c0_seq1”.

The quality of the transcript assembly was checked by Transrate version 1.0.3 [110]. Transrate was also used to compare transcripts to available oak reference genomes by read mapping through Conditional Reciprocal Best BLAST with default cut-off value of 1e-5. Candidate coding regions within assembled transcripts were predicted by Transdecoder software version 5.1.0 [111]. Transcriptome completeness was checked by Benchmarking Universal Single-Copy Orthologs (BUSCO) version 3 based on the plant ortholog database (embryophyta_odb9) [112]. Reads were mapped back to the transcriptome assembly with bowtie2 v2.2.1 using the –sensitive parameter.

### Functional annotation, pathway identification and gene expression analysis

Gene ontology (GO) functional classification of the transcriptome assembly was carried out using the Blast2GO program [113] based on the NCBI non-redundant (nr) protein sequences by fast-BLASTX [114] with an E-value cut-off of 1e-5 as well as EBML-EBI InterProScan (IPS) database. Gene ontology [115] terms were obtained for each putative transcript from both BLAST and IPS outputs. WEGO [116] was used to examine the GO terms among the annotated putative transcripts. The EC numbers were retrieved through GO-EnzymeCode Mapping feature of Blast2GO software.

### Identification, annotation and enrichment analyses of differentially expressed genes

For differential gene expression of ozone exposure, only data from the second ozone experiment was used for analysis; this experiment had individually barcoded replicates and high depth of reads generated by the HiSeq instrument. To obtain raw read counts for each putative transcript per library HTSeq version 0.6.1 [117] was used. The raw count matrix were provided to the edgeR version 3.6 bioconductor package [118] to distinguish differentially expressed genes (DEGs) between treatment and control groups. Briefly, normalization by the trimmed mean of M-values (TMM) method was calculated to adjust count reads. Normalized factors, the counts per million (CPM), were used in common, trended and tag-wise dispersion analyses by Cox-Reid profile-adjusted likelihood (CR) method. Finally, to determine significant DEGs negative binomial general linearized model (GLM) likelihood ratio was tested based on the model (treatment*time), where treatment was ozone concentrations, and time was time-points for each treated sample. Genes were considered significantly differentially expressed based on adjusted *p*-value < 0.05 [119] and |log2 (fold change)| > 1. Consensus DEGs detected by edgeR package were visualized by Venny version 2.1 [120] and their results were used in further annotation and enrichment analyses.

GO enrichment analysis of DEGs was carried out by agriGO v2 [121] with the significant DEGs of each model as the foreground dataset and all putative transcripts as the background reference. The statistical parameters used for identification of overrepresented GO terms were Fisher’s exact test, adjusted for multiple testing by FDR with a cut-off value at the significance level of 0.05. Statistical enrichment of DEGs within constructed pathways based on Kyoto Encyclopedia of Genes and Genomes (KEGG) database was tested by KEGG Orthology-Based Annotation System (KOBAS) program [122].

### Time-series analysis of differentially expressed genes

The analysis of DEGs over time was analyzed by STEM using the log fold-change of DEGs (O_3_-treated versus control) among the three time points, where all samples from the same time point were combined. The parameters in STEM were adjusted as follows: maximum unit change in model profiles between time points set to 1; maximum output profiles number set to 50. The clustered profiles with *p*-value < 0.05 were defined as significant profiles. The enriched clusters were further analyzed by KOBAS to determine their GO terms and KEGG pathways, of which biological function of profiles with adjusted p-value < 0.05 were considered significant.

### Weighted gene co-expression network analysis

TMM-normalized gene expression values were used in an R package, WGCNA [123], to identify modules containing co-expressed genes. After removal of genes with zero normalized counts, one-step network construction and module detection were performed using unsigned block-wise-module comprised of at least 100 genes per module. A consensus gene expression profile for each module was represented by the module eigengene that was calculated through first principal component analysis. The module-factor relationship was obtained through Pearson correlation coefficient. The top hub gene, i.e. the gene with the highest connectivity, for each module was identified with the WGCNA package.

## Supplementary information

**Additional File 1: Table S1.** Number of reads and bases produced for each RNA library used in assembly.

**Additional File 2: Figure S1.** E-value distribution of northern red oak sequence hits obtained in BLAST analysis against nr database, and metrics of transcriptome assembly obtained by Transrate.

**Additional File 3: Figure S2.** Second-tier GO terms assigned to northern red oak transcripts.

**Additional File 4: Figure S3.** Image of northern red oak leaf after exposure to 125 ppb ozone for 28 days. Symptoms of toxicity in NRO leaves are visible as inter-vein red stippling and small lesions.

**Additional File 5: Table S2.** Unique and common DEGs in ozone stress.

**Additional File 6: Figure S4.** Enriched GO terms for ozone stress.

**Additional File 7: Table S3.** Enriched KEGG pathways of ozone stress.

**Additional File 8: Table S4.** Profiles of clustered DGEs over time-series analysis by STEM.

**Additional File 9: Table S5.** Coexpression network data, including module membership for all genes, top hub gene for each module, and enriched GO and KEG terms for the modules most strongly correlated to experimental factors

## Data Availability

All RNA-Seq data are available in the NCBI Sequence Read Archive database under BioProject accession number PRJNA273270. The reference transcriptome sequences are available on the Hardwood Genomics Project website (https://www.hardwoodgenomics.org/Transcriptome-assembly/1963023).

## Abbreviations

ABCG 11: ABC transporter family G member 11
BP: Biological process
CC: Cellular component
DEG: Differential expressed gene
EC: Enzyme commission
EDS1: Enhanced disease susceptibility 1
ETI: Effector-trigger immunity
FDR: False discovery rate
GGDP: Geranylgeranyl pyrophosphate
GO: Gene ontology
HMG-CoA: 3-Hydroxy-3-methylglutaryl-coenzyme A
HR: Hypersensitive response
HSP: Heat shock protein
IPP: Isopentenyl pyrophosphate
IPS: Interproscan
KEGG: Kyoto encyclopedia of genes and genomes
MEP: Methylerythritol phosphate
MF: Molecular function
MVA: Mevalonic acid
NRO: Northern red oak
O_3_: ozone
ORF: Open reading frame
PPB: Part per billion
PR: Pathogenesis-related protein
PTI: Pathogen-associated molecular pattern-triggered immunity
ROS: Reactive oxygen species
TCA: Citrate cycle
