# Supplementary material for: Transcriptome profiles of Quercus rubra responding to increased O3 stress
Source: BMC Genomics. 2020 Feb 14;21:160. doi: 10.1186/s12864-020-6549-5 (PMC7023784; doi:10.1186/s12864-020-6549-5)
Supplement: Supplementary file 2 — Additional File 2: Figure S1. E-value distribution of northern red oak sequence hits obtained in BLAST analysis against nr database, and metrics of transcriptome assembly obtained by Transrate. [file 12864_2020_6549_MOESM2_ESM.pdf]

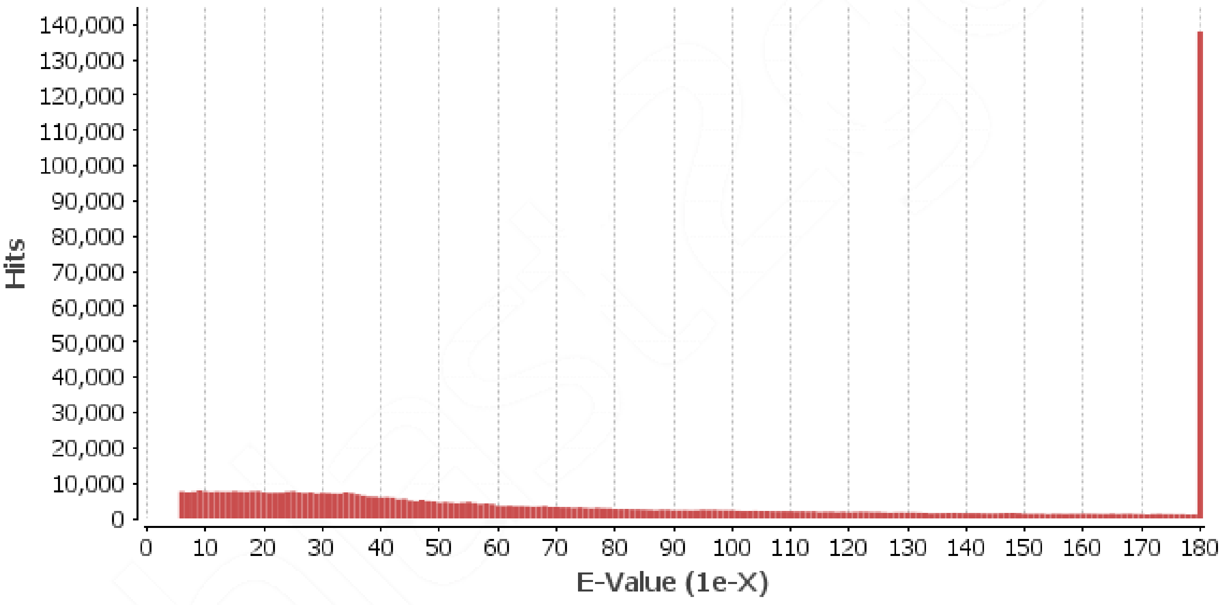

Evalue distribution of sequence hits.

.

Contig metrics of transcriptome assembly of northern red oak generated by Transrate.

**Contig Info.**	**Contig metrics**
Number (N) of sequences	52,662
Smallest	201
Largest	7,661
N of bases	40,981,774
Mean sequence length	778.2
N over 1k	13,786
N90	313
N50	1,244
N10	2,870
GC Percentage	41.6%
