# Supplementary figures and images for: Transcriptome profiles of Quercus rubra responding to increased O3 stress
Source: BMC Genomics. 2020 Feb 14;21:160. doi: 10.1186/s12864-020-6549-5 (PMC7023784; doi:10.1186/s12864-020-6549-5)

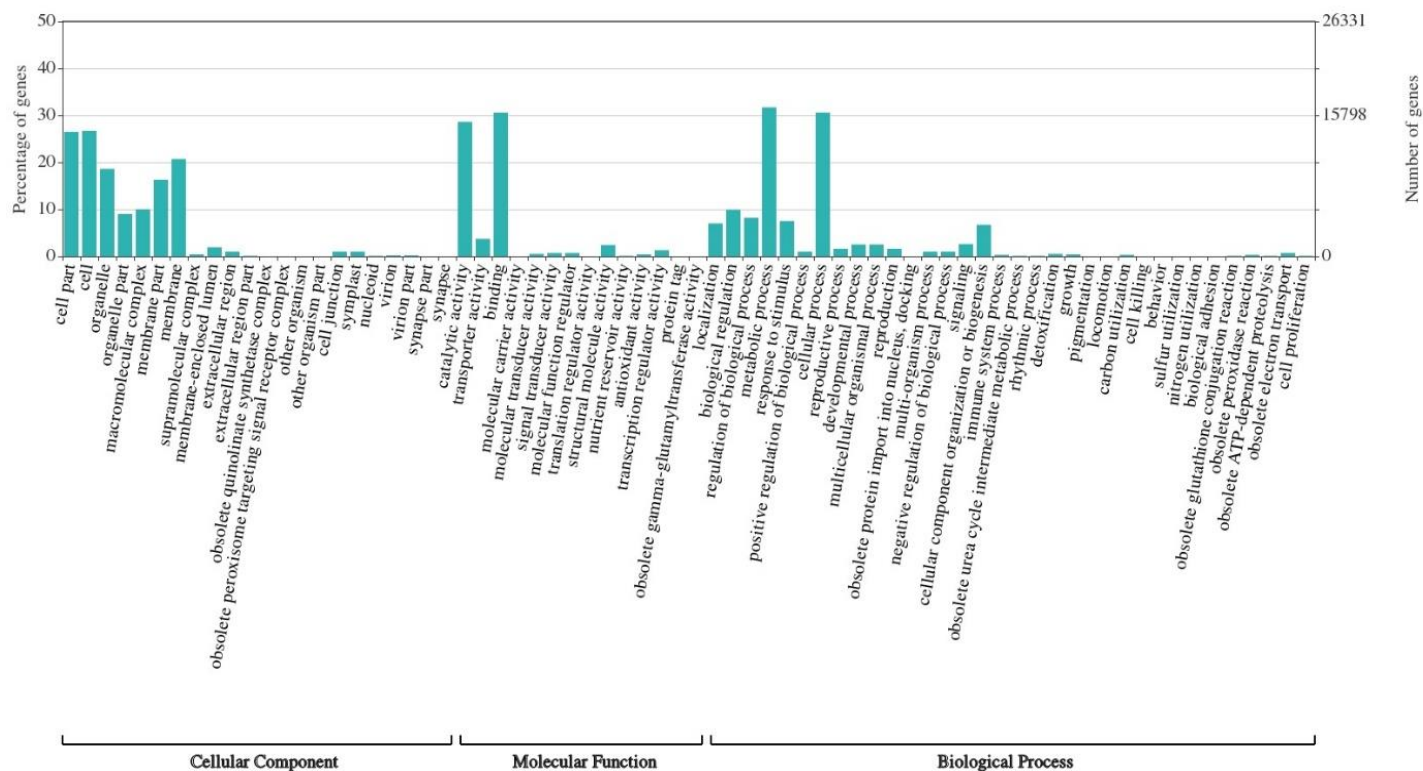

Supplement: Supplementary file 3 — Additional File 3: Figure S2. Second-tier GO terms assigned to northern red oak transcripts. [file 12864_2020_6549_MOESM3_ESM.pdf]

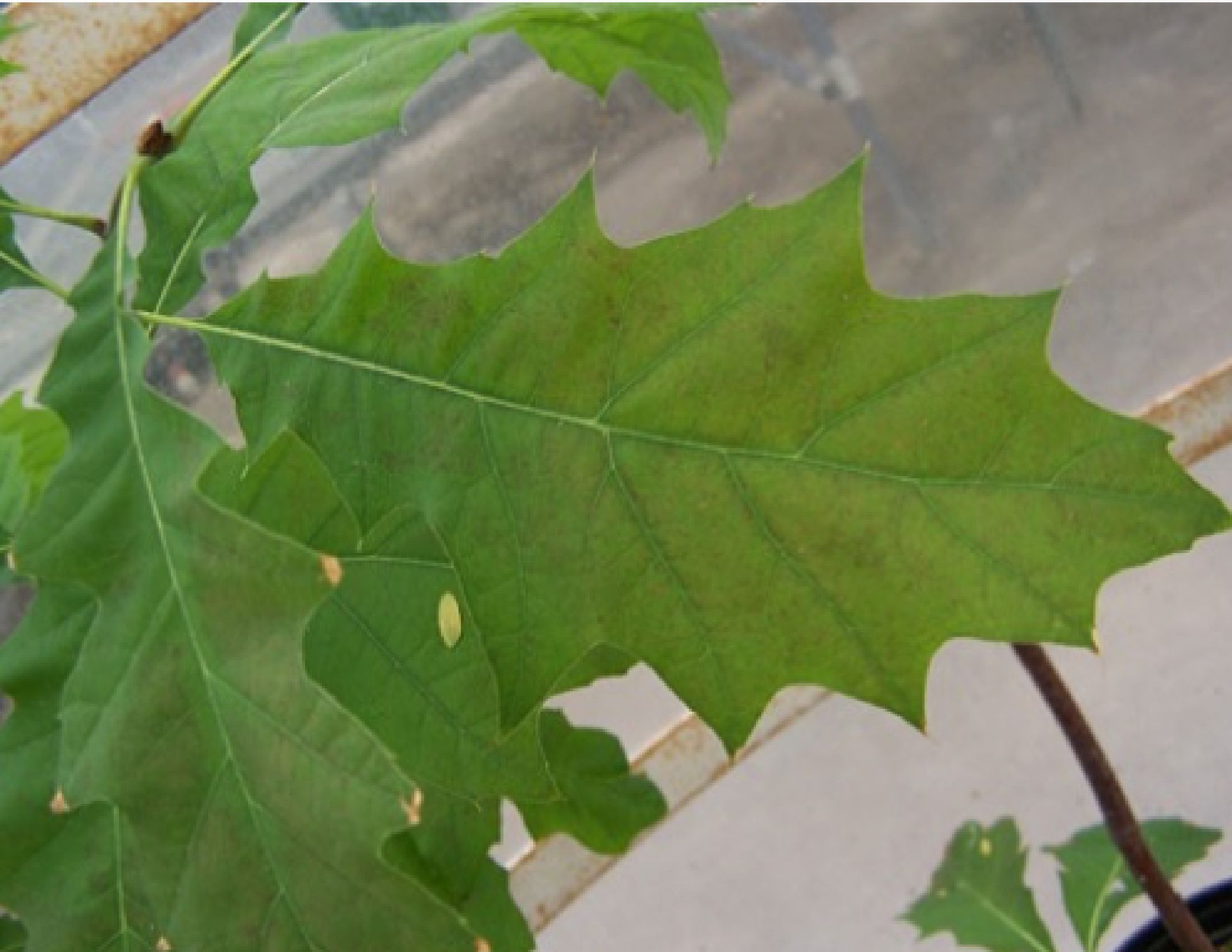

Supplement: Supplementary file 4 — Additional File 4: Figure S3. Image of northern red oak leaf after exposure to 125 ppb ozone for 28 days. Symptoms of toxicity in NRO leaves are visible as inter-vein red stippling and small lesions. [file 12864_2020_6549_MOESM4_ESM.pdf]
