# Supplementary material for: Transcriptome profiles of Quercus rubra responding to increased O3 stress
Source: BMC Genomics. 2020 Feb 14;21:160. doi: 10.1186/s12864-020-6549-5 (PMC7023784; doi:10.1186/s12864-020-6549-5)
Supplement: Supplementary file 6 — Additional File 6: Figure S4. Enriched GO terms for ozone stress. [file 12864_2020_6549_MOESM6_ESM.pdf]

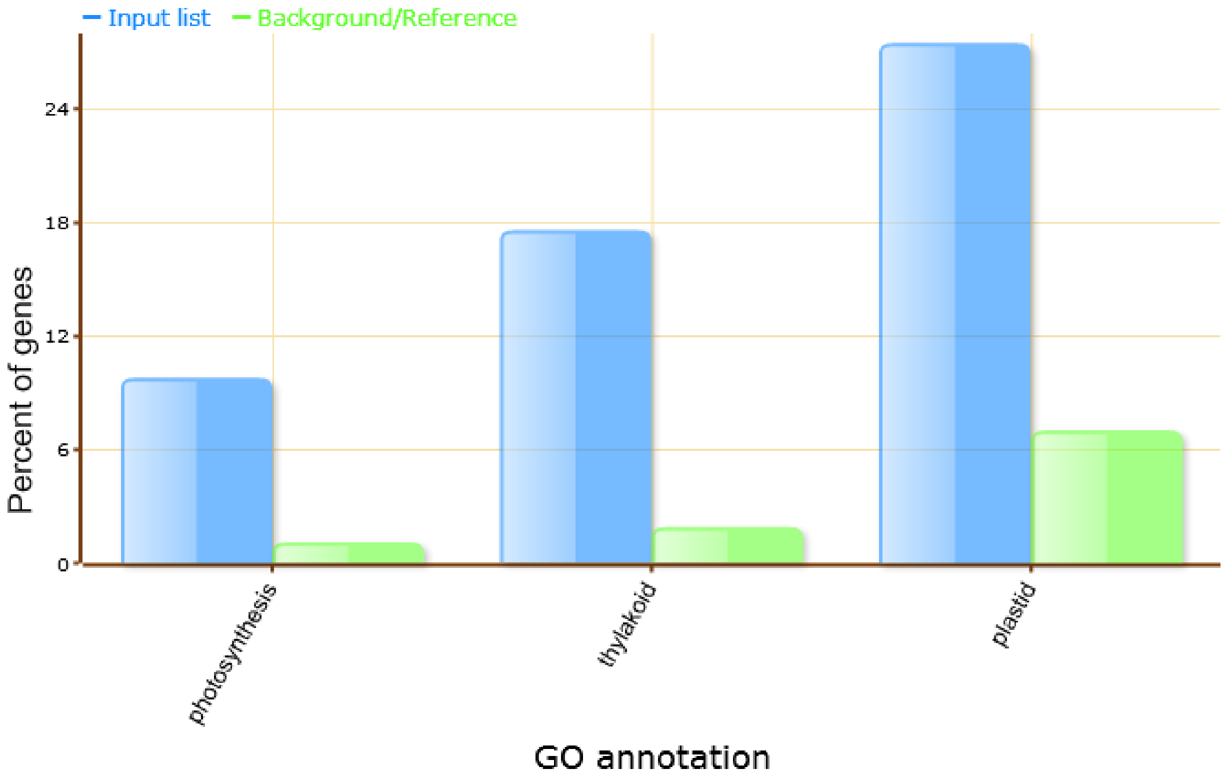

Second-tier GO terms identified only at 14day in downregulated DEGs exposed to 225ppb of ozone

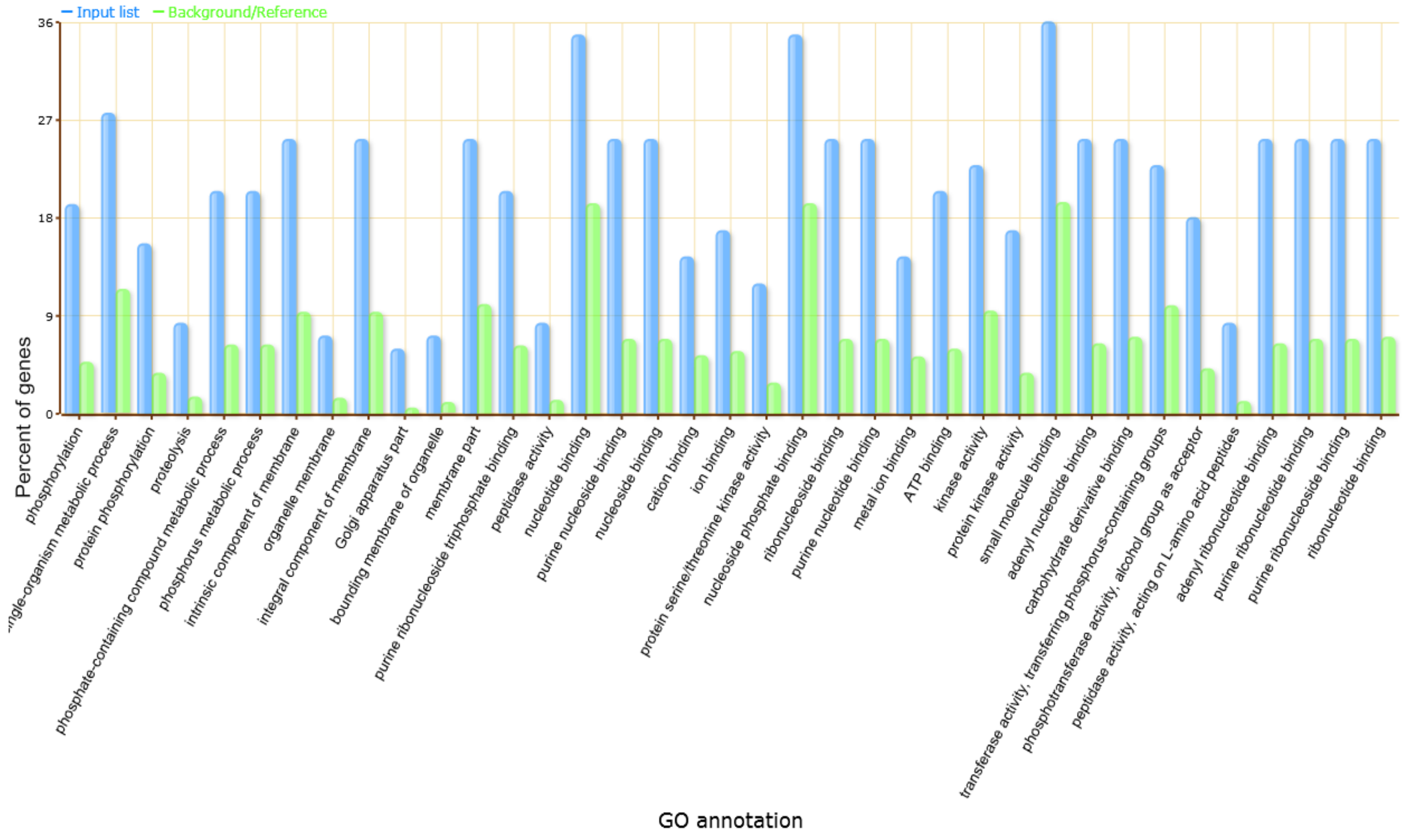

Second-tier GO terms identified only at 14day in upregulated DEGs exposed to 225ppb of ozone

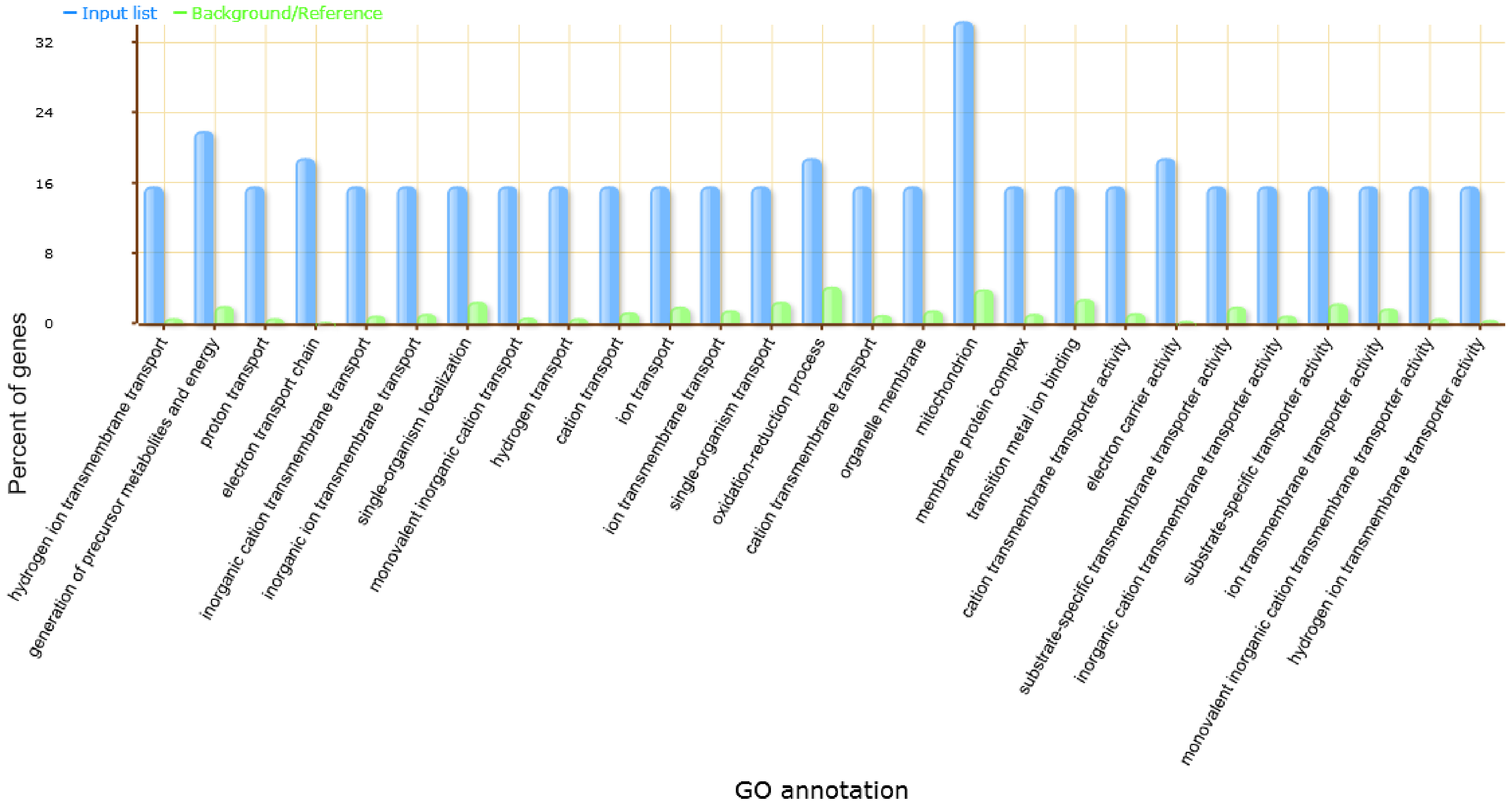

Second-tier GO terms identified only at 28day in upregulated DEGs exposed to 80ppb of ozone

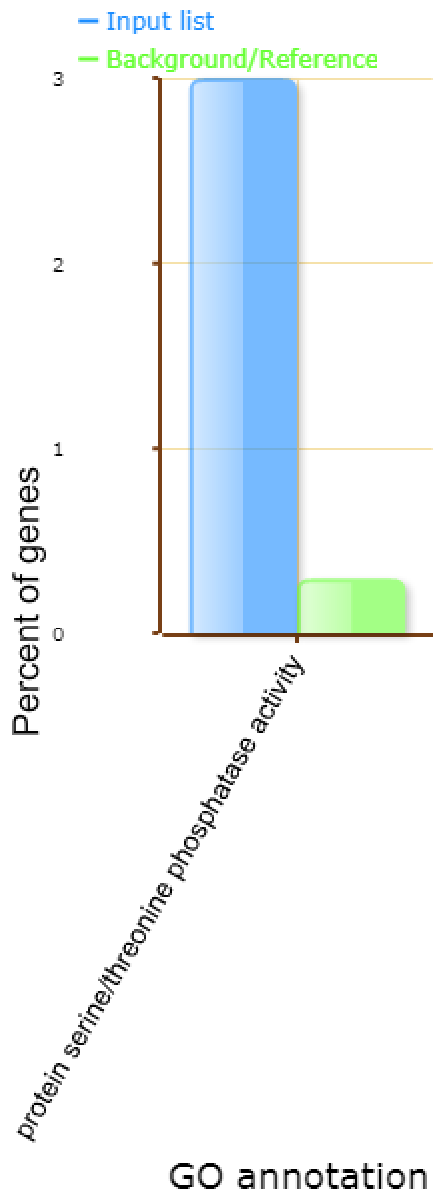

Second-tier GO terms identified only at 28day in upregulated DEGs exposed to 125ppb of ozone

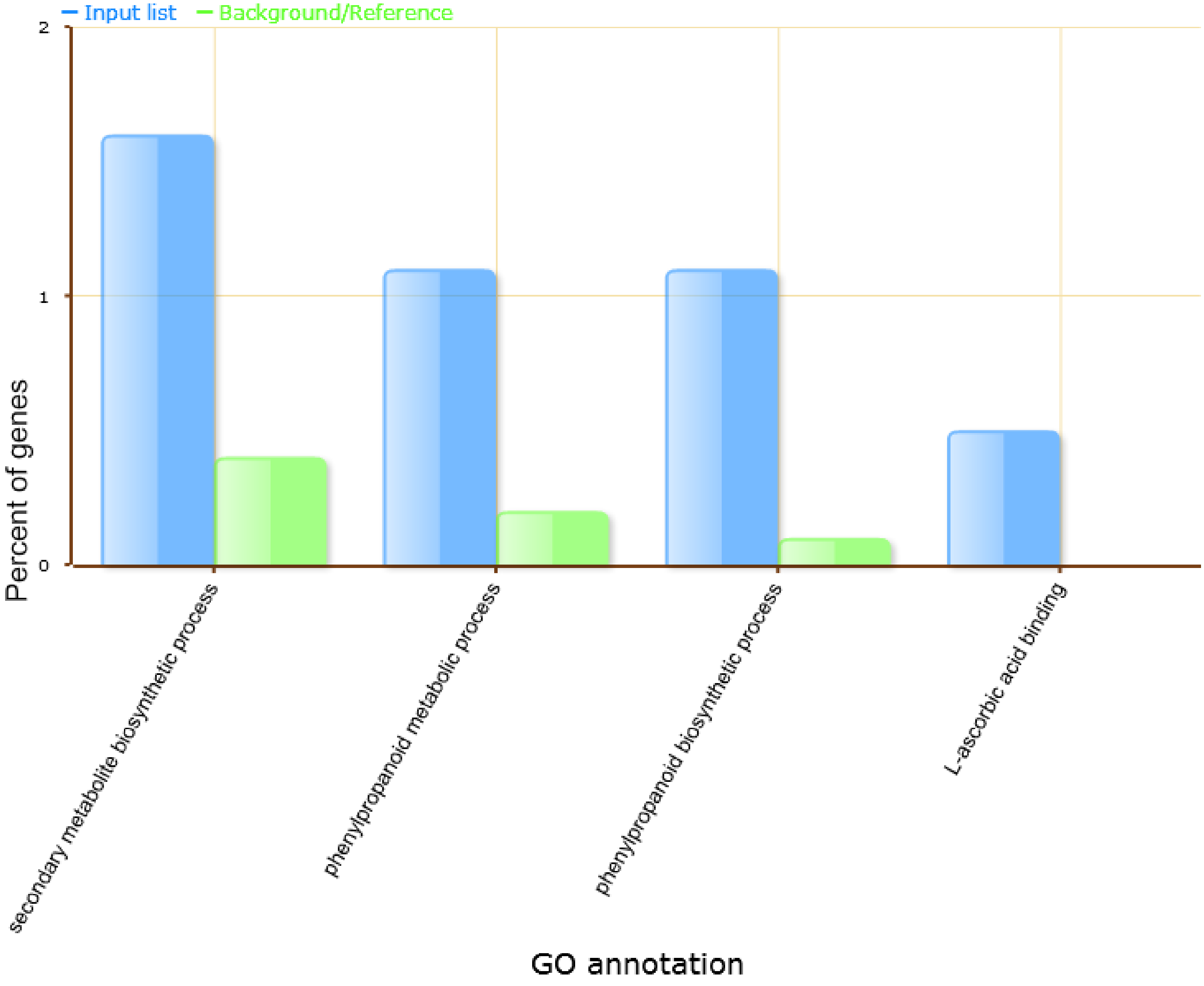

Second-tier GO terms identified only at 28day in upregulated DEGs exposed to 225ppb of ozone

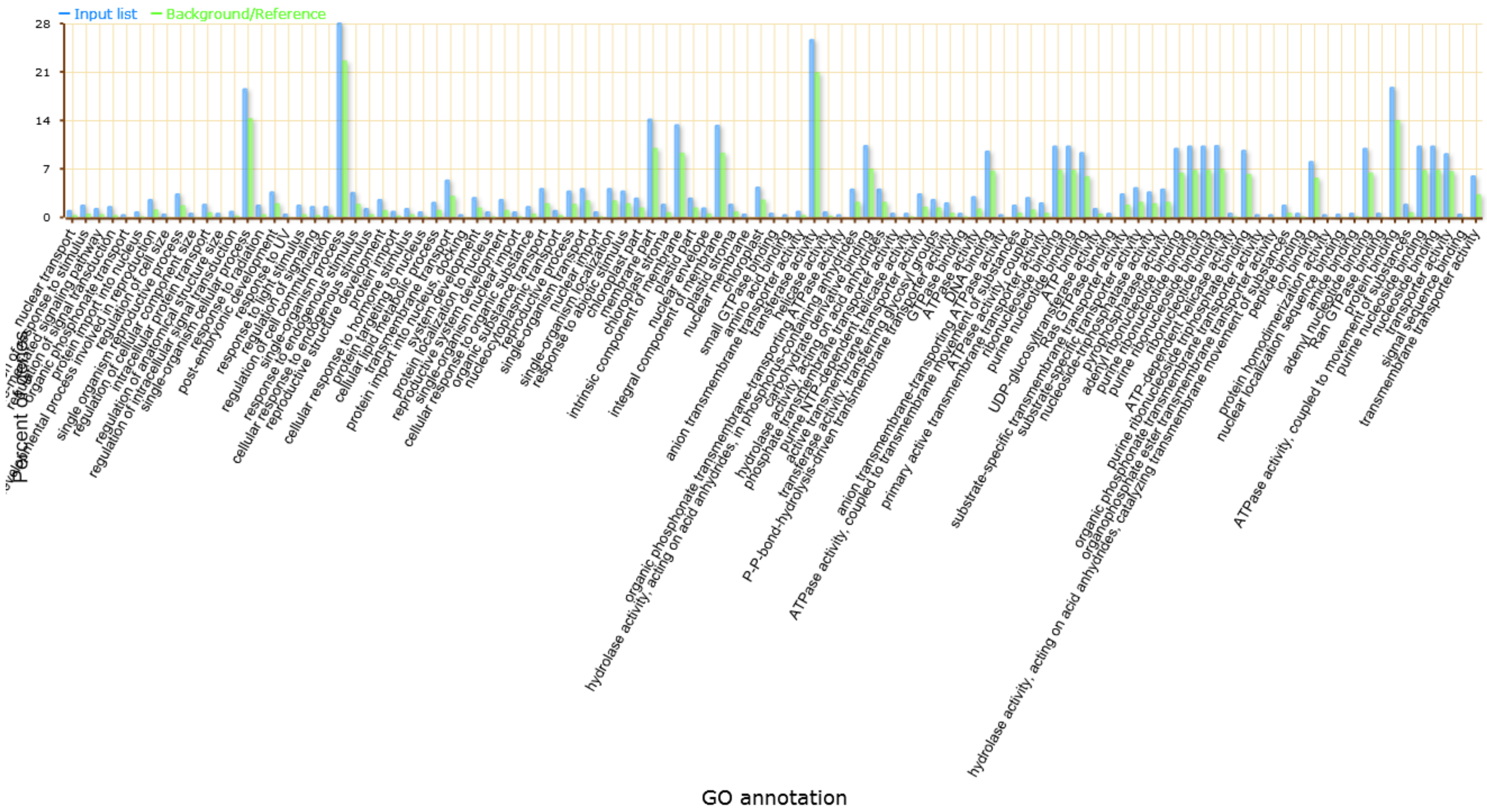

Second-tier GO terms identified only at 28day in downregulated DEGs exposed to 225ppb of ozone

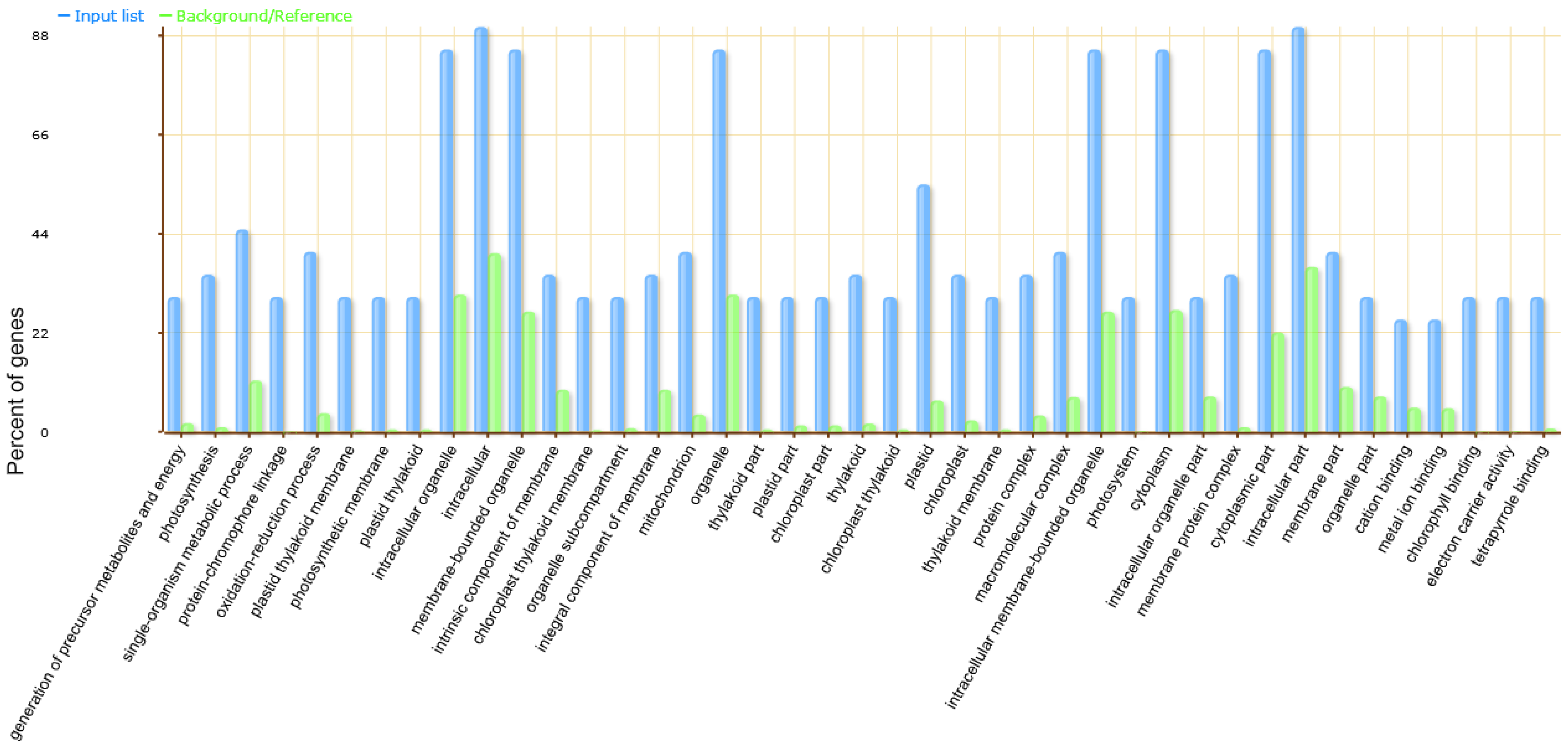

Second-tier GO terms shared between 14day&28day in upregulated DEGs exposed to 80ppb of ozone

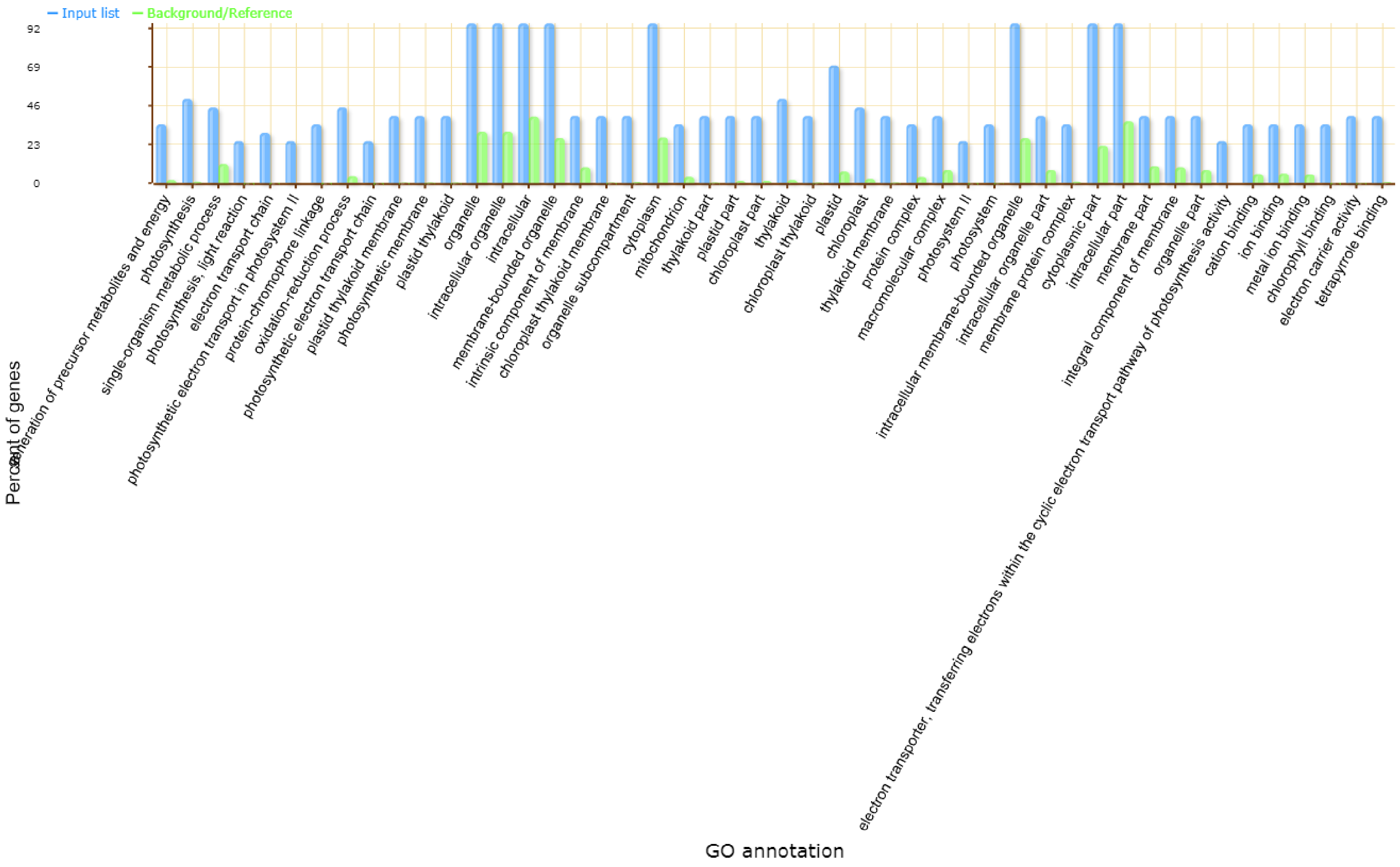

Second-tier GO terms shared between 7hour&14day in upregulated DEGs exposed to 125ppb of ozone

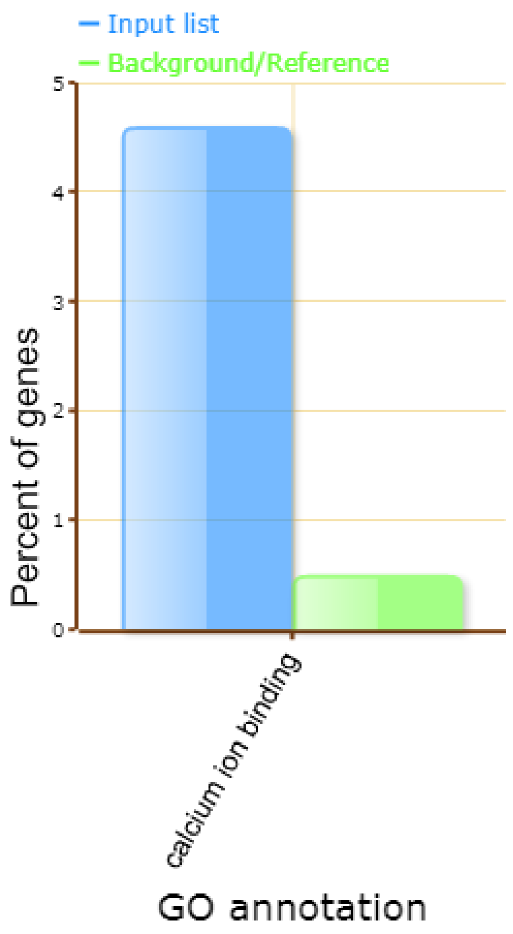

Second-tier GO terms shared between 14day&28day in upregulated DEGs exposed to 225ppb of ozone

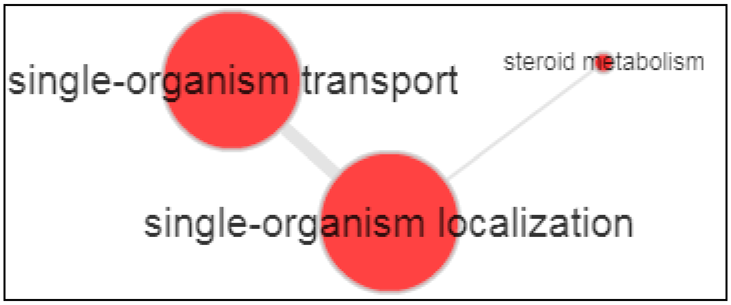

Second-tier GO terms in downregulated DEGs exposed to 225ppb of ozone

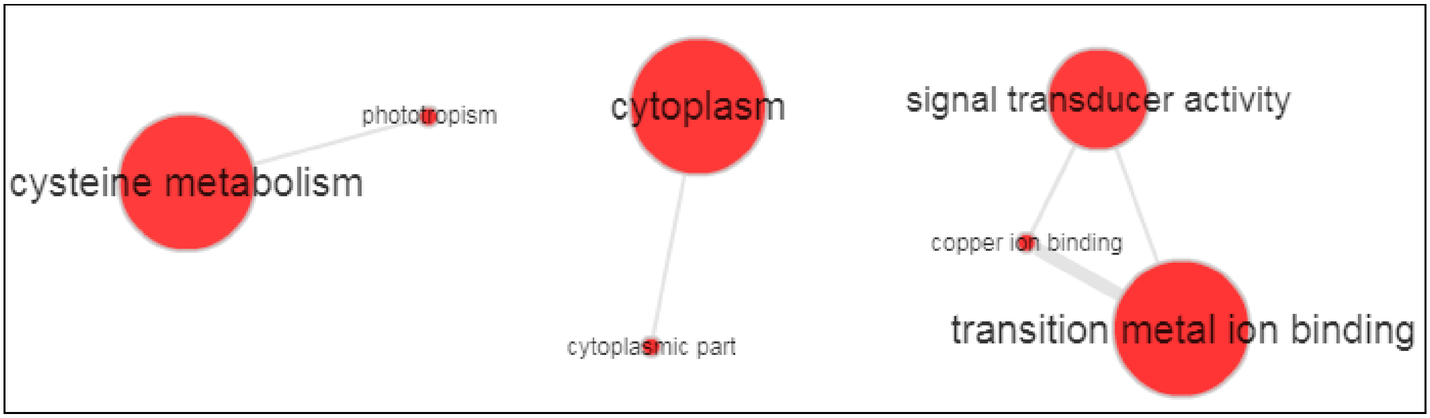

Second-tier GO terms in upregulated DEGs exposed to 225ppb of ozone
